# The role of Sox6 in zebrafish muscle fiber type specification

**DOI:** 10.1186/s13395-014-0026-2

**Published:** 2015-01-27

**Authors:** Harriet E Jackson, Yosuke Ono, Xingang Wang, Stone Elworthy, Vincent T Cunliffe, Philip W Ingham

**Affiliations:** A*STAR Institute of Molecular and Cell Biology, Proteos, 61 Biopolis Drive, Singapore, 138673 Republic of Singapore; Bateson Centre, University of Sheffield, Western Bank, Sheffield, S10 2TN UK; Lee Kong Chian School of Medicine, Nanyang Technological University, Proteos, 61 Biopolis Drive, Singapore, 138673 Republic of Singapore; Department of Medicine, Imperial College, South Kensington Campus, London, SW7 2AZ UK

**Keywords:** zebrafish, muscle, fiber type, Sox6, troponin, myosin, spinal curvature

## Abstract

**Background:**

The transcription factor Sox6 has been implicated in regulating muscle fiber type-specific gene expression in mammals. In zebrafish, loss of function of the transcription factor Prdm1a results in a slow to fast-twitch fiber type transformation presaged by ectopic expression of *sox6* in slow-twitch progenitors. Morpholino-mediated Sox6 knockdown can suppress this transformation but causes ectopic expression of only one of three slow-twitch specific genes assayed. Here, we use gain and loss of function analysis to analyse further the role of Sox6 in zebrafish muscle fiber type specification.

**Methods:**

The GAL4 binary misexpression system was used to express Sox6 ectopically in zebrafish embryos. Cis-regulatory elements were characterized using transgenic fish. Zinc finger nuclease mediated targeted mutagenesis was used to analyse the effects of loss of Sox6 function in embryonic, larval and adult zebrafish. Zebrafish transgenic for the GCaMP3 Calcium reporter were used to assay Ca2+ transients in wild-type and mutant muscle fibres.

**Results:**

Ectopic Sox6 expression is sufficient to downregulate slow-twitch specific gene expression in zebrafish embryos. Cis-regulatory elements upstream of the *slow myosin heavy chain 1 (smyhc1)* and *slow troponin c* (*tnnc1b*) genes contain putative Sox6 binding sites required for repression of the former but not the latter. Embryos homozygous for *sox6* null alleles expressed *tnnc1b* throughout the fast-twitch muscle whereas other slow-specific muscle genes, including *smyhc1,* were expressed ectopically in only a subset of fast-twitch fibers. Ca2+ transients in *sox6* mutant fast-twitch fibers were intermediate in their speed and amplitude between those of wild-type slow- and fast-twitch fibers. *sox6* homozygotes survived to adulthood and exhibited continued misexpression of *tnnc1b* as well as smaller slow-twitch fibers. They also exhibited a striking curvature of the spine.

**Conclusions:**

The Sox6 transcription factor is a key regulator of fast-twitch muscle fiber differentiation in the zebrafish, a role similar to that ascribed to its murine ortholog.

**Electronic supplementary material:**

The online version of this article (doi:10.1186/s13395-014-0026-2) contains supplementary material, which is available to authorized users.

## Background

Vertebrate skeletal muscle is composed of distinct fiber types that differ in their physiological and metabolic properties; Type I or slow-twitch fibers have a low contraction velocity but are rich in mitochondria and are therefore more efficient at using oxygen to generate ATP, resulting in a high endurance capability. Type II or fast-twitch fibers, by contrast, are more suited to generating short burst of strength or speed, but they fatigue more rapidly than slow-twitch fibers due to their high contraction velocity. Although largely genetically determined, the fiber type composition of muscles is also partially adaptive; endurance training by long distance runners, for instance, can increase their proportion of Type I fibers through conversion of Type IIa fibers. Most studies of the control of muscle fiber type in mammals have focused on their activity dependent diversity and plasticity [1]; by contrast, rather less is known about the allocation of myoblasts to distinct fates during embryonic development.

The zebrafish provides a highly tractable model to study vertebrate fiber type specification, as the embryonic myotome shows a discrete temporal and spatial separation of fiber type ontogeny that facilitates genetic analysis of its development [2]. Zebrafish myogenesis begins prior to somite formation with the activation of the myogenic regulatory factors (MRFs), *myoD* and *myf5* [3-6] The cells closest to the notochord, the so-called adaxial cells [7], are the first myoblasts to be specified and begin to differentiate prior to somitogenesis in response to notochord-derived Hedgehog (Hh) signals [4,8-13]. Most adaxial cells elongate and migrate radially outward to form a subcutaneous layer of mononucleated slow-twitch muscle fibers named superficial slow-twitch fibers (SSF) [7]. A specialized subpopulation of adaxial cells, the muscle pioneers (MPs) are characterized by their expression of the Engrailed transcription factors and retain their medial location to form the horizontal myoseptum that subdivides the myotome into dorsal (epaxial) and ventral (hypaxial) compartments [7,14,15]. The bulk of the myotome comprises the fast-twitch fibers, which begin their differentiation in the wake of the migrating slow-twitch fibers [4,16]. The fast muscle progenitors mature and fuse with each other to form a multinucleated array of syncytial fibers [13].

The Sry transcription family member Sox6 has been implicated in muscle fiber type specification in both mice and fish. Mice mutant for *sox6* display an increase in slow-specific gene expression and a concomitant decrease in the expression of fast-twitch specific genes [17,18], suggesting that Sox6 normally functions to promote the fast-twitch differentiation program and repress slow-specific gene expression in fetal muscle fibers. Consistent with this, ChIPseq analysis has revealed the direct interaction of Sox6 with the regulatory elements of slow-specific genes in mice [19,20]. In zebrafish embryos lacking activity of the Prdm1a transcription factor, adaxial cells differentiate into fast-twitch fibers, a transformation that is accompanied by the ectopic expression of *sox6*. Transient knockdown of Sox6 mediated by morpholino antisense oligonucleotides is sufficient to suppress this transformation, suggesting that similar to its role in mouse, Sox6 normally acts to repress slow-twitch gene expression in zebrafish [21]. Surprisingly, however, while *tnnc1b* is de-repressed in the fast fibers of Sox6 morphant embryos, no ectopic *smyhc1* expression was observed. This could reflect an incomplete inactivation of Sox6 function achieved by morpholinos or indicate a different pathway of repression and/or activation of *smyhc1*. Here, we have used targeted overexpression and mutagenesis of the *sox6* gene to explore further its role in zebrafish muscle fiber type specification. Our findings confirm and extend the results of our previous transient knock-down studies and imply that Sox6 is not the sole mediator of slow-twitch gene repression.

## Methods

### Ethics statement

The research described in this paper uses the zebrafish as an alternative to mammalian experimental models. Adult zebrafish were raised and maintained under internationally accepted conditions in the Institute of Molecular and Cell Biology (IMCB) Zebrafish Aquarium Facility, accredited by the Animal and Veterinary Authority (AVA) of Singapore. All experimental procedures were performed in compliance with and approved by the Agency for Science Technology and Research (A*STAR) Biological Resource Centre Institutional Animal Care and Use Committee (IACUC Project #110638). Most experimentation and analysis was restricted to the first 5 days postfertilization (dpf). Homozygous mutant fish were regularly monitored, and any showing signs of distress were humanely euthanized following accepted protocols.

### Zebrafish strains and husbandry

Adult fish were maintained on a 14 hour light/10 hour dark cycle at 28°C in the AVA (Singapore) certificated IMCB Zebrafish Facility. Previously described zebrafish strains used were: *Tg(smyhc1:GFP)*^*i104*^ [22]; *prdm1a*^*nrd*^ [23]; *Tg*(*prdm1:GFP)*^*i106*^ [22] and *Tg(actin1β:GAL4)*^*i269*^ line [24].

### Generation of UAS:Sox6-GFP

The *sox6* ORF was amplified by PCR and cloned into pDONR221 to make pME-sox6, and then recombined with p5E-UAS, p3E-GFP and pDestTol2pA by gateway cloning. The resultant UAS:sox6-GFP plasmid was injected into one-cell stage embryos with *tol2* mRNA to generate the *Tg(UAS:sox6-GFP)*^*i295*^ line.

### Real-time PCR analysis

Real-time PCR was performed on a Bio-Rad (Hercules, CA, USA) iQ5 real-time PCR detection system using KAPA SYBR FAST qPCR Kit (KAPA Biosystems, Wilmington, MA, USA), according to the manufacturer’s protocols. Primer sets were designed for *smyhc1* (forward, CCTGGTGTCTCAGTTGACCA; reverse, TGTGCCAGGGCATTCTTT), *tnnc1b* (forward, GCAAGATCGACTACGACGAG; reverse, AGGCAGCATTGGTTCAGG), *mylz2* (forward, CAGGTTCACCGCAGAGGA; reverse, TTCGTTTTCTTGATTCCAAGG), and *b-actin* (forward, TGGCATTGCTGACCGTATGC; reverse, GTCATGGACGCCCATTGTGA). Real-time PCR was performed with cDNA samples synthesized from 3μg of total RNA from approximately 50 embryos. Relative mRNA expression levels were calculated based on cycle threshold and amplification efficiency of each primer set. Expression of *b-actin* gene was used as internal control for normalization.

#### *Generation, selection and genotyping of* sox6 *mutant alleles*

Plasmids encoding zinc-finger nucleases (ZFN) specific for the zebrafish *sox6* gene were purchased from Sigma-Aldrich (St. Louis, MO, USA). The zinc-finger nuclease was designed so that it targeted the sequence CTGGCACGCCAAcagcaAGAGCAGGTGAGAATGTG, which is present in both isoforms of Sox6, upstream of the HMG box. Capped polyadenylated RNA from each plasmid was produced by *in vitro* transcription, and a range of doses was injected into one-cell stage zebrafish embryos.

G0 adults derived from embryos injected with ZFN capped RNA were incrossed and their progenies (G1) individually genotyped by PCR using the *sox6* ZF forward primer (GGGTGCAGGGTTGTGAAGTG) and the *sox6* ZF reverse primer (ATACATGCACATTACTGCAGGTG) followed by Sanger sequencing using the *sox6* ZF seq primer (CTTCCTTCTTCCATTTTGTTC). Two alleles, *sox6*^*i291*^ and *sox6*^*i292*^, were isolated, each of which introduces a premature stop codon into the open reading frame that are predicted to encode truncated forms of the protein.

#### *Generation of* tnnc1b:eGFP *transgenic line*

An eGFP-SV40pA-FRT-Kn-FRT recombineering targeting cassette and red recombineering system in EL250 cells were used to insert eGFP with an SV40 polyadenylation site at the *tnnc1b* ATG start site in BAC ZC137P17 [25], which has at least 25 kb of upstream sequence and 200 kb of downstream sequences from the *tnnc1b* gene. This BAC was further modified by the addition of Tol2 sites and a stable line, *Tg(BACtnnc1b:EFGP)*^*i293*^*,* was generated using Tol2-mediated transgenesis [26]. Deletion derivatives of the BAC were made by further targeted recombination events. Downstream deletions were made by targeting the iTol2-Amp-iTol2 cassette into the eGFP modified BAC but designing the homology arms so that the right arm had homology to the sequence 1 kb downstream from the *tnnc1b* stop codon and the left arm had homology to the other side of the chloramphenicol marker resulting in the chloramphenicol gene being replaced by the ampicillin and a large amount of downstream sequence deleted from the BAC. Upstream deletions were made by targeting a construct containing a kanamycin resistance gene to different loci in the BAC so differing amounts of upstream sequence were recombined out of the BAC. Successful deletion of upstream and downstream sequences was confirmed by PCR. Smaller constructs containing the eGFP reporter sequence were PCR-amplified out of the BAC and cloned into the pDB739 vector that contains Tol2 sites [27] (gift of Steve Ekker, Mayo Clinic, Rochester, MO, USA).

A β-globin minimal promoter reporter vector was generated by excising β-globin-eGFP-polyA from βg-eGFP-SP72 [28] using EcoRI and cloned into the same site of pDB739 [27]. Potential enhancer fragments were cloned into this vector.

### Site-directed mutagenesis

To mutate potential Sox6 binding sites in the *smyhc1:GFP* promoter, the promoter proximal to the NotI site in the *Tg(smyhc:GFP)*^*i104*^ construct [22] was subcloned. Five potential Sox6 binding sites were identified manually and mutated from AACAAT to AAAAAT using the Stratagene (La Jolla, CA, USA) Quickchange Multi Site-Directed Mutagenesis Kit. After mutagenesis the mutated promoter was subcloned back into the full *smyhc1:GFP* plasmid and stable transgenic lines were created.

To mutate potential Sox6 binding sites in the *tnnc1b:GFP* promoter, the sequence 2.5 kb upstream of the start site of *tnnc1b,* as well as the first intron, was analyzed manually to identify possible Sox6 binding sites. Sites were mutated by designing forward and reverse primers that had approximately 10-bp homology to the sequence 5’ of the potential Sox6 binding site and 30-bp homology to the sequence 3’ of the Sox6 site, with a 4-bp mismatch (ACAAT mutated to AGGG) in the core sequence of the Sox6 binding site. After PCR using these primer pairs and iProof DNA polymerase (Biorad, Hercules, CA, USA), 1μl of DpnI restriction enzyme was added to the reaction to digest the methylated plasmid template. After a 2-hour incubation at 37°C, the sample was PCR-purified (AxyPrep PCR clean-up kit Axygen, Union City, CA, USA) and transformed. Sequences were analyzed using Lasergene SeqMan (DNA STAR Madison, WI, USA). Successfully mutated plasmids were injected into zebrafish embryos, and stable transgenic lines were created for each construct.

### *In situ* hybridization and immunofluorescence

*In situ* hybridization of whole embryos and cryosections was performed as previously described [29,30]. Fluorescent *in situ* hybridization utilized anti-DIG peroxidase and fluorescence substrate Cy5 tyramide signal amplification (TSA, Perkin Elmer, Waltham, MA, USA) and was performed according to the manufacturer’s protocol. Probes used were made from plasmids *smyhc1* [22], *tnnc1b* [21], *myh7b* [31]*, ryr1a* [32] and *prox1a* [33]. *mylz10, tpm2, tnnt1 and tnni1a* were cloned from zebrafish cDNA into a pGEM-T vector (Promega, Madison, WI, USA). Probes were made by linearizing and transcribing with the appropriate restriction enzyme and polymerase respectively. Images were captured using the Carl Zeiss (Oberkochen, Germany) AXIO Zeiss Imager M2 and the AXIO Vision 4.7.2 software. To analyze the effects of the misexpression of Sox6, embryos were collected from a *UAS:sox6-GFP;actin:GAL4* incross and separated based on their Sox6-GFP expression; embryos expressing strong Sox6-GFP were collected into one tube, and embryos expressing no Sox6-GFP were separated into another tube. Reactions were carried out in separate tubes, with the same number of embryos in each and were stained for exactly the same length of time.

### Monitoring Ca2+ flux using GCaMP3

The 2 dpf larvae were mounted in 2% low-melting agarose gel containing 50 μM blebbistatin, and muscle contraction was induced by 40 mM pentylenetetrazole (PTZ) as previously described [34]. The *smyhc1:GCaMP3* construct was generated by replacing the *gfp* coding sequence of the 9.7kb *smyhc1:gfp construct* [22] with *GCaMP3* coding sequence and used to generate a transgenic line, Tg(*smyhc1:GCaMP3)*^*i280*^. Fast-twitch specific transient transgenics were generated by injecting the *mylz2:GCaMP3* construct [34]. A total of 1 nl of DNA plasmid was injected into the one-cell stage embryo at 20 ng/μl. Fluorescent change was measured every 70 to 90 ms with Olympus (Tokyo, Japan) FV-1000 Fluoview confocal microscopy, and average fluorescence intensity was analyzed using Olympus FV10-ASW software.

### Immunohistochemistry

Antibody staining was performed as previously described [22,31] at the following dilutions: mAb F310 (anti-fast myosin light chain; DSHB) at 1:50; mAb F59 (anti-slow myosin heavy chain; DSHB) at 1:50; Rabbit anti-eGFP (Torrey Pines) 1:500; and rabbit anti-zebrafish Sox6 [31] 1:500. The following secondary antibodies were used: anti-rabbit IgG-488 (Invitrogen) 1:100 and anti-mouse IgG-546 (Invitrogen) 1:1000. Specimens were imaged with an Olympus Fluoview confocal microscope. Images were acquired using Olympus FV10-ASW software and analyzed using ImageJ software (http://rsbweb.nih.gov/ij/).

## Results

### Misexpression of Sox6 in adaxial cells represses slow-twitch specific gene expression

**Figure 1:**
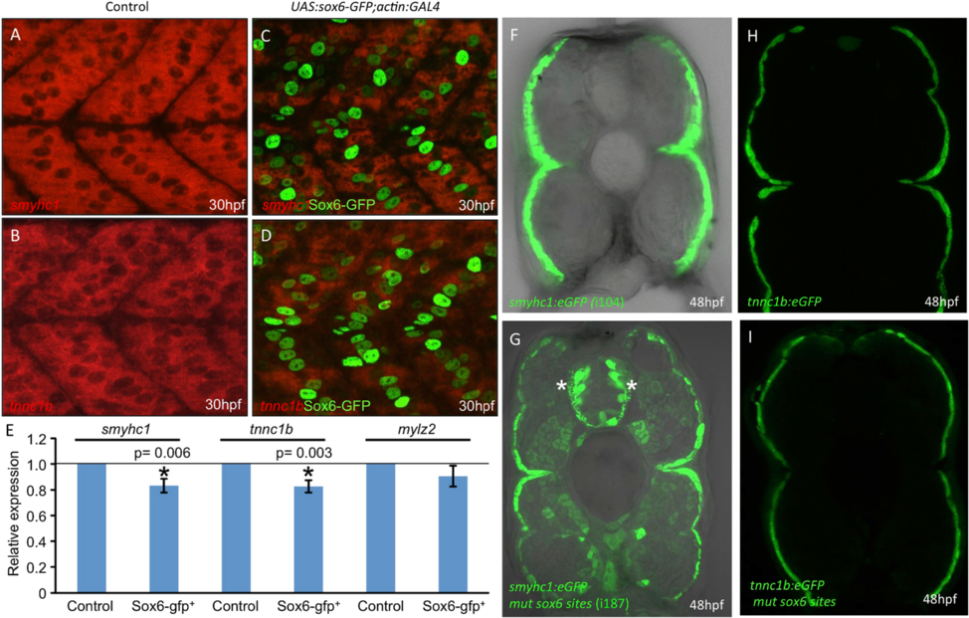
**Control of slow-twitch specific gene expression by Sox6** ***.*** **(A,B)** Fluorescent *in situ* hybridization of 30 hours postfertilization (hpf) wild-type embryos revealing accumulation of *smyhc1* and *tnnc1b* transcripts throughout the mononucleated slow-twitch muscle; **(C,D)** transgenic embryos expressing Sox6-GFP in slow-twitch fibers (green nuclei) hybridized under identical conditions to the wild-type controls **(A,B)** showing reduced levels of *smyhc1* and *tnnc1b* transcripts; **(E)** graphical representation of data pooled from three independent qPCR analyses of *smyhc1*, *tnnc1b* and *mylz2* transcription in control and *UAS:sox6-GFP;actin:GAL4* (*sox6-gfp* ^*+*^ *)* embryos at 30 hpf. Student’s two-tailed, unpaired t-test, error bars represent standard deviation, control n = 3, *sox6-gfp* ^*+*^ n = 3, asterisks indicate significance; **(F)** Cross section through a trunk somite of a 48 hpf *smyhc1:GFP* transgenic embryo; note that expression is restricted to the superficially located slow-twitch muscle fibers; **(G)** similar preparation of an embryo transgenic for the same *smyhc1:GFP* reporter gene in which the putative Sox6 binding sites have been mutated: note the ectopic expression in many fast-twitch muscle fibers as well as in prominent clusters of neurons in the neural tube (asterisks). **(H)** Cross section through a trunk somite of a 48 hpf *tnnc1b:GFP* transgenic embryo: expression is similarly restricted to the slow-twitch fibers. **(I)** Similar preparation of a 48 hpf embryo transgenic for the same reporter in which the putative Sox6 binding sites have been mutated: note the absence of ectopic expression of this transgene.

To investigate whether Sox6 activity is sufficient to repress slow-twitch specific gene expression, we generated a transgenic line in which a Sox6-GFP fusion protein can be driven under the control of the GAL4 transcriptional activator. Fish carrying this transgene, Tg(*UAS:sox6-GFP)*^*i295*^*,* were crossed with those carrying a previously described muscle specific *actin:GAL4* transgene [24]. Fluorescent *in situ* hybridization of embryos that were 30-hours postfertilization (hpf) indicated that expression of *tnnc1b and smyhc1* was reduced in the Sox6-GFP expressing fibers (Figure 1A-D). Similarly, expression of the slow-specific genes *troponin t1* (*tnnt1), troponin i1a (tnni1a), myosin light chain 10 (mylz10), tropomyosin 2* (*tpm2)* and the *ryanodine receptor 1a* (*ryr1a)* also appeared to be downregulated in embryos expressing Sox6-EGFP (Additional file 1: Figure S1). To confirm these findings, we performed qPCR analysis of *smyhc1* and *tnnc1b* transcription in Sox6-GFP and control embryos. In both cases, we found a modest but statistically significant reduction in transcript levels; by contrast, levels of *mylz2* transcript did not differ significantly between Sox6-GFP expressing and control embryos (Figure 1E). Embryos with strong GFP expression in the head muscle at 4 dpf were sorted from GFP-negative embryos. *In situ* hybridization for *tnnc1b* transcripts revealed no effect of ectopic Sox6 activity on *tnnc1b* expression in the head muscles (Additional file 2: Figure S2). These findings are in line with the previous demonstration that ectopic expression of Sox6 can repress Prox1a expression in adaxial cells [21]. Taken together, they demonstrate that Sox6 activity is sufficient to inhibit the expression of slow-twitch muscle genes in the embryonic trunk muscle of the zebrafish.

### *cis-*acting sequences mediate Sox6 dependent repression of *tnnc1b* and *smyhc1*

Previous studies have identified cis-regulatory regions of the *smyhc1* gene that drive reporter gene expression specifically in adaxial cells and slow-twitch fibers [22,35]. Five potential Sox6 sites were identified in this upstream sequence and mutated; transgenic lines carrying the mutated reporter construct showed ectopic expression of the reporter in fast-twitch fibers of embryos at 48 hpf (Figure 1G and H), implying that these sites are required for the lineage specific repression of *smyhc1*. The ectopic eGFP expression was not observed in every fast-twitch fiber but was restricted to a subset of fast-twitch fibers.

**Figure 2:**
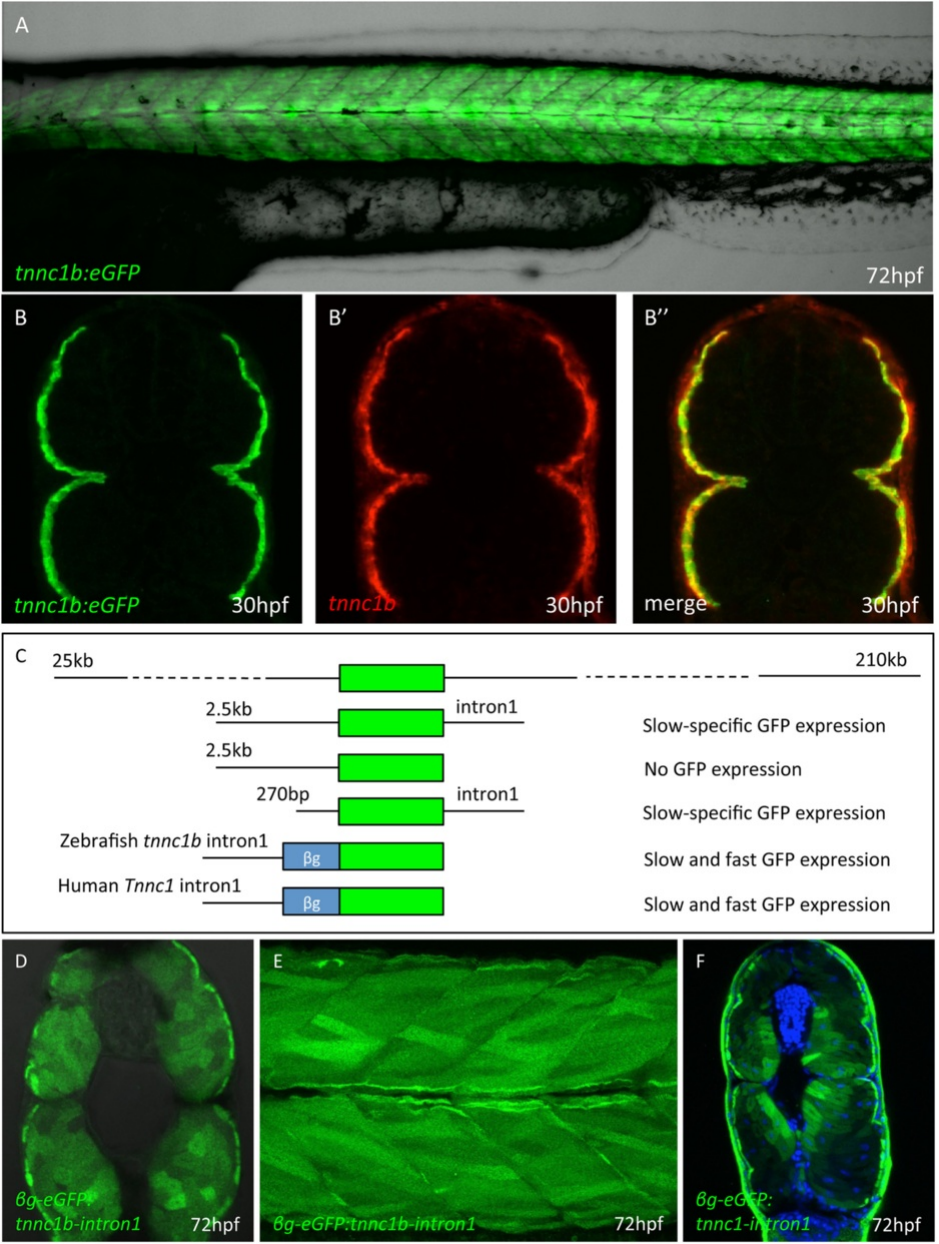
**Defining the** ***tnnc1b*** **cis regulatory region. (A)** Trunk region of a *Tg(BACtnnc1b:eGFP*)^*i293*^ larva 72 hpf showing reporter gene expression specifically in the slow-twitch muscle. **(B)** Cross section through the mid-trunk region of a 30 hpf *Tg(BACtnnc1b:eGFP*)^*i293*^ embryo showing reporter gene expression restricted to the superficial layer of muscle fibers: **(B’)** the same section showing endogenous *tnnc1b* mRNA assayed by fluorescent *in situ* hybridization. **(B”)** Merged images showing that the reporter faithfully recapitulates the expression pattern of the endogenous gene. **(C)** Schematic representation of deletion derivatives of the *BACtnnc1b:eGFP* reporter construct (Green box = eGFP); the expression patterns observed in transgenic lines carrying these construct is indicated on the right. **(D)** Cross section and **(E)** optical sagittal section of a 72 hpf transgenic larva showing expression of eGFP throughout the myotome driven by the first intron of the zebrafish *tnn1cb* intron and a mammalian βglobin minimal promoter. **(F)** Cross section of a 72 hpf transgenic embryo in which expression of eGFP is driven by the first intron of the human *slow troponin c* gene. The strong signal in the skin in is a staining artefact and was not present in live embryos.

To explore the basis of *tnnc1b* regulation further, an eGFP reporter cassette was inserted into a *tnnc1b* containing BAC by homologous recombination [25] such that the eGFP ATG start site replaced the *tnnc1b* ATG start site. The resulting *tnnc1b:eGFP* reporter gene recapitulated the endogenous pattern of *tnnc1b* expression in transgenic embryos, with expression restricted to the slow-twitch muscle and overlapping precisely with the endogenous *tnnc1b* transcript (Figure 2A and B). To define the *cis-*regulatory regions more precisely, several reporter constructs containing differing lengths of the *tnnc1b* upstream and downstream sequence were generated and their activities assayed in transgenic embryos (Figure 2C). This deletion analysis revealed that the first intron of *tnnc1b* is essential for the expression of the gene. This putative intron 1 enhancer element was cloned downstream of a β-globin minimal promoter vector to assay its activity. Interestingly, eGFP expression was observed throughout the myotome in both the fast-twitch and slow-twitch fibers (Figure 2D and E) indicating that it acts as a general muscle-specific enhancer. Previous analyses identified a skeletal muscle enhancer in the first intron of the mouse *Tnnc1* gene and this shows high conservation with the human *Tnnc1* gene [36]. The first intron of human *Tnnc1* was cloned into the β-globin minimal promoter vector and found to drive eGFP expression in both the slow-twitch and fast-twitch muscle in stable transgenic zebrafish embryos (Figure 2F). It follows that the slow-twitch specificity of the *tnnc1b* gene is mediated by repressor elements upstream of its promoter.

Five potential Sox6 binding sites were identified in the 2.5-kb upstream sequence + intron 1. Progressive mutation of these sites had no effect on reporter gene expression in transgenic embryos (Figure 1I; Additional file 3: Figure S3). Further deletion of the *tnnc1b* promoter revealed that just 270 bp upstream of the transcription start site together with the first intron was sufficient to drive slow-specific expression in zebrafish embryos (Figure 2C). This 270-bp upstream region contains no canonical Sox6 sites, though two sites with an imperfect match (0.85 identity) were detected. Potential sites for many other transcription factors were also identified by in silico analysis (data not shown), but none of these stood out as an obvious candidate for mediating slow-twitch fiber specific expression.

### Sox6 represses only a subset of slow-specific genes in fast-twitch fibers

**Figure 3:**
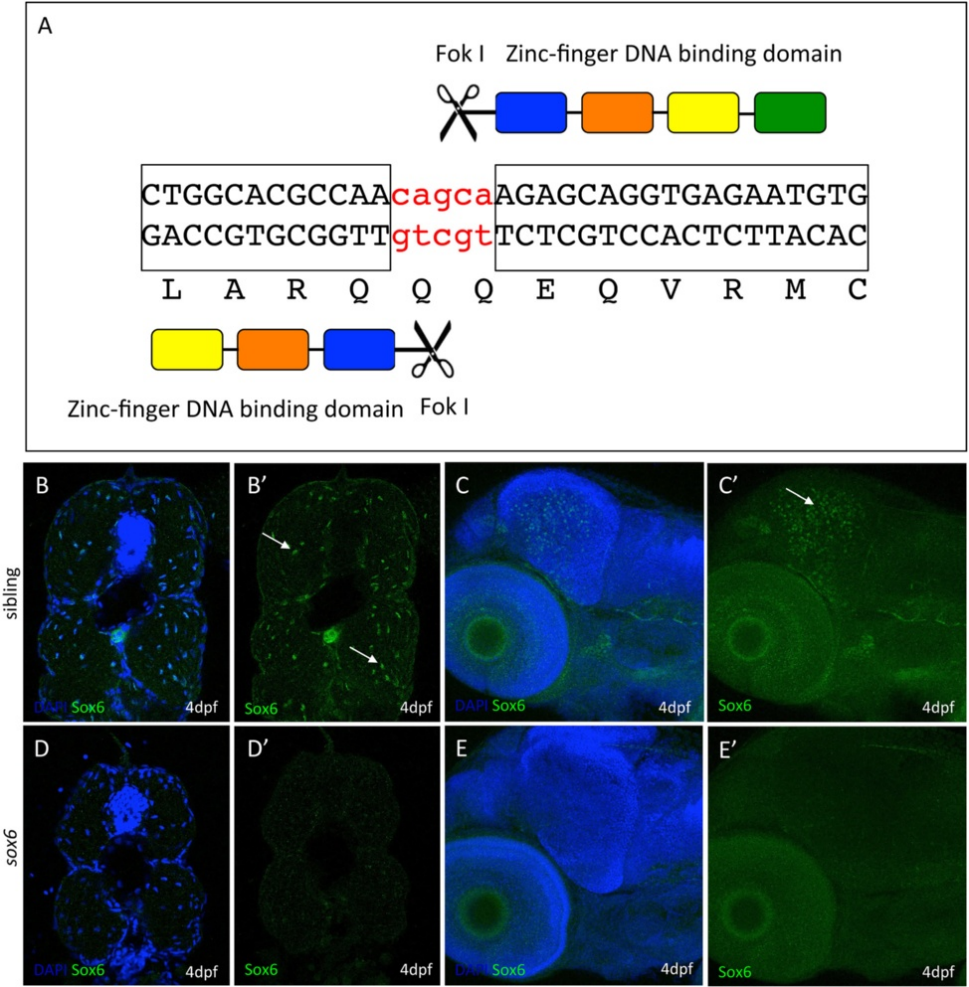
**Targeted mutagenesis of the zebrafish** ***sox6*** **locus. (A)** Schematic representation of the zinc-finger nuclease designed to target genomic sequences in exon 8, upstream of the HMG box, of the zebrafish *sox6* gene. **(B-E)** Immunohistochemical detection of Sox6 protein using a Sox6-specific antibody; Sox6 protein is detectable in the nuclei of fast muscle fibers (arrows) **(B and B’)** and the optic tectum (arrow) **(C and C’)** of wild-type embryos. In *sox6* homozygous mutants, by contrast, no signal is detected in either the muscle **(D and D’)** or the optic tectum **(E and E’)**, indicating the successful generation of a null mutant. (Blue signal = DAPI).

Expression of *sox6* can first be detected in the somites at the ten-somite stage [31] when it is restricted to fast-twitch fiber progenitors and excluded from adaxial cells. To investigate Sox6 function further, mutant alleles were created using zinc-finger nuclease (ZFN) mediated targeted mutagenesis [37] (Figure 3A). Homozygous mutant embryos lacked full-length Sox6 protein in both fast-twitch fibers and in the forebrain, as assayed by whole-mount immunohistochemistry using a Sox6-specific antibody (Figure 3B-E).

**Figure 4:**
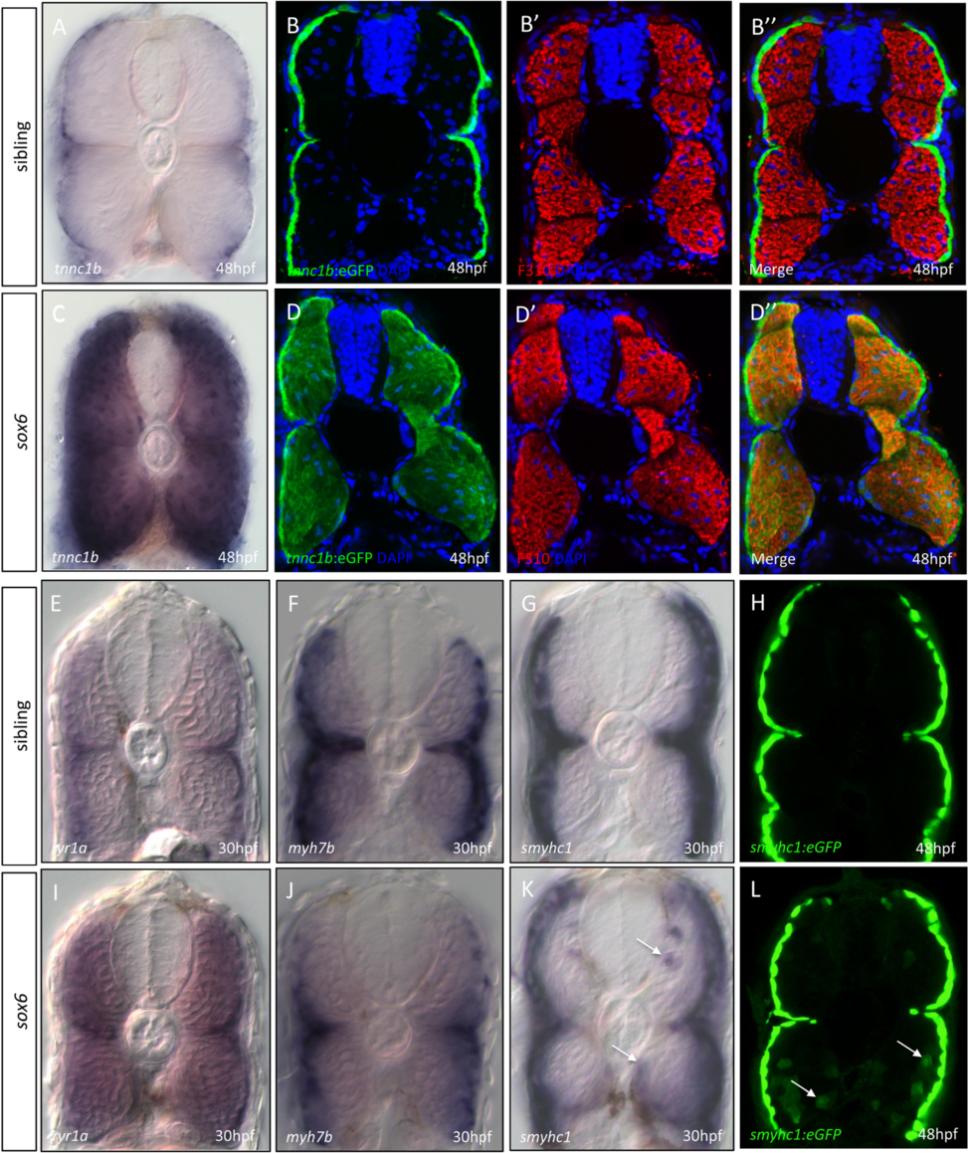
***tnnc1b*** **is ectopically expressed in fast-twitch fibers of homozygous** ***sox6*** **mutants.** Endogenous *tnnc1b* expression **(A)** and *tnnc1b:eGFP* reporter expression (green) **(B)** is restricted to slow-twitch fibers of wild-type embryos at 48 hours postfertilization (hpf) and excluded from fast-twitch fibers (**B’**:red) as shown in merged image **(B”)**. In *sox6* mutants, endogenous *tnnc1b* **(C)** and the *tnnc1b:eGFP* reporter **(D)** are expressed in both slow and fast-twitch fibers (**D’**: red) as shown in merged image (**D”**). Expression of *ryr1a* is restricted to slow-twitch fibers in wild type **(E)** but ectopically expressed in the fast-twitch fibers in *sox6* mutant embryos at 30 hpf (**I**). By contrast, *myh7b* expression remains restricted to slow-twitch fibers in *sox6* mutants (cf. **F** and **J**). Slow-twitch specific expression of the endogenous *smyhc1* **(G)** and the *smyhc1:GFP* reporter **(H)** in wild-type embryos. In *sox6* mutants **(K,L)**, expression is largely similarly restricted but ectopically expressed in a few fast fibers (arrows).

**Figure 5:**
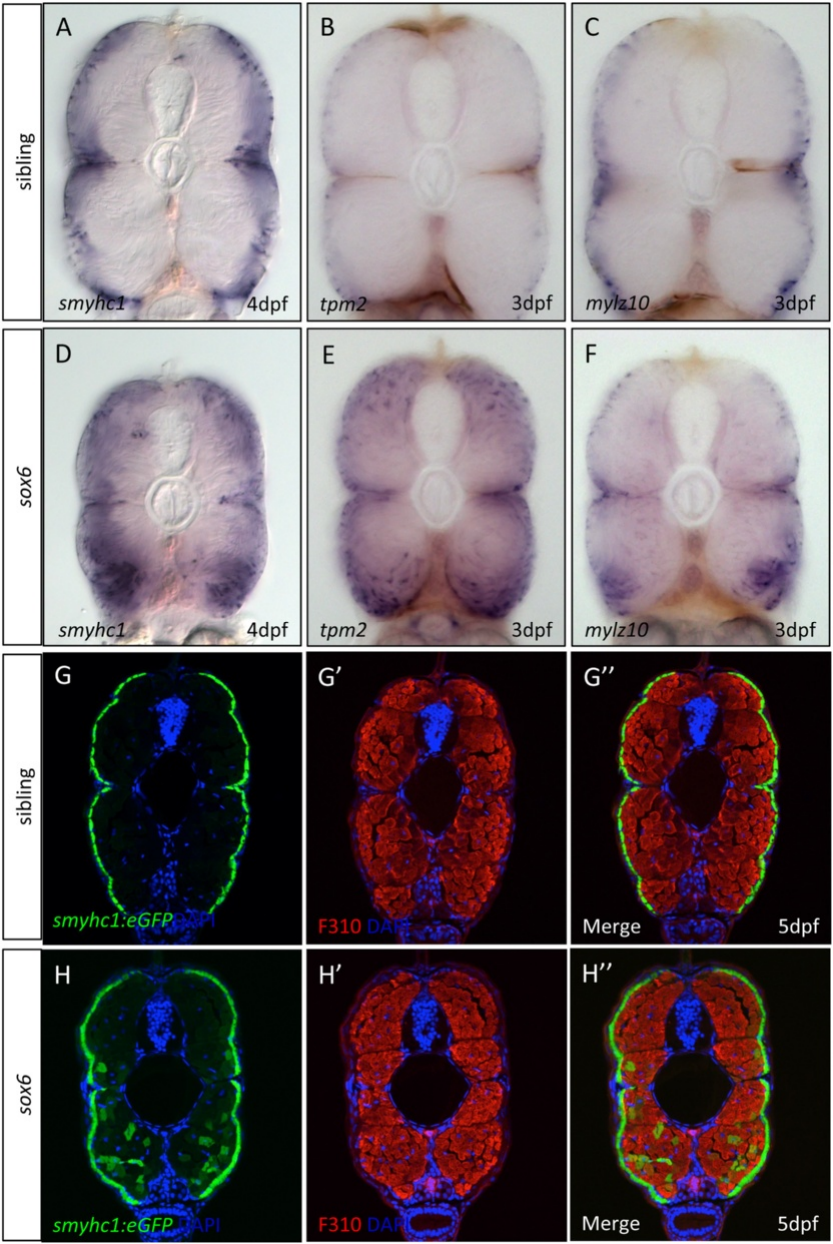
**Partial de-repression of slow-twitch genes in** ***sox6*** **mutant larvae. (A-C)** *smych1, tpm2* and *mylz10* remain restricted to the slow-twitch fibers in wild-type larvae at 3 or 4 days postfertilization (dpf). **(D-F)** All three genes are ectopically expressed in some fast-twitch fibers in *sox6* mutants by 3 or 4 dpf. **(G-G”)** *smyhc1:GFP* expression (green) is excluded from fast-twitch fibers (red) in 5 dpf wild-type larvae.** (H-H”)** In *sox6* mutant larvae at 5 dpf, a subset of fast-twitch fibers ectopically express the reporter gene **(H).** The expression of the fast-twitch myosin revealed by mAb F310 (red) in the fast domain remains unaffected in *sox6* mutants (cf **G’** and **H’**).

In line with previous morpholino-based analyses [21], ectopic *tnnc1b* expression was observed throughout the fast-twitch domain in the trunk and tail of *sox6* mutant embryos from approximately 22 hpf onward (Figure 4A and C), but not in craniofacial fast-twitch muscles (Additional file 4: Figure S4). Similarly, the *tnnc1b:eGFP* transgene was ectopically expressed in the fast-twitch fibers in *sox6* homozygotes (Figure 4B and D). Like *tnnc1b,* the slow-specific *ryr1a* (Figure 4E and I) and *tnnt1* (Additional file 5: Figure S5A and F) were also ectopically expressed throughout the fast-twitch domain in *sox6* mutants at 30 hpf. Interestingly, this was not the case for all slow-twitch muscle genes: the expression patterns of *myh7b* (Figure 4F and J) *tnni1a, mylz10, tpm2* and *prox1a* (Additional file 5: Figure S5B-E and G-J), were unaffected in *sox6* mutant embryos at 30 hpf. Ectopic expression of *smyhc1* could be detected in a small number of fast-twitch fibers by 30 hpf in *sox6* mutants, both by *in situ* hybridization for the endogenous transcript (Figure 4G and K) and by reporter gene expression (Figure 4H and L). This pattern of ectopic expression resembled that of the mutant *smyhc1:eGFP* reporter construct described above (cf. Figure 1H). By 3 dpf, fast fibers positive for *tpm2* and *mylz10* expression could also be readily detected in *sox6* mutant larvae (Figure 5B,C,E and F). Similarly, more fast fibers expressed *smyhc1* in *sox6* mutants by 4 to 5 dpf (Figure 5A,D,G and H).

In contrast to the aberrant expression of slow-specific genes, expression of fast myosin light chain proteins, detected with the F310 antibody (Figure 5 G’ and H’), and expression of fast specific troponin subunit, assayed by WISH (Additional file 6: Figure S6), were unaffected in *sox6* homozygotes.

### Loss of Sox6 rescues slow-specific gene expression in Prdm1 mutants

**Figure 6:**
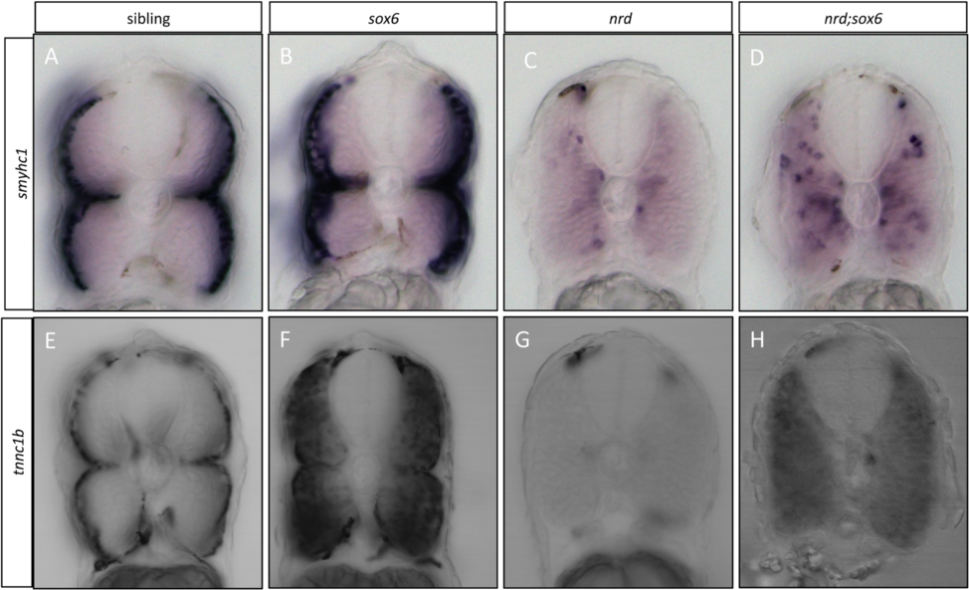
**Elimination of** ***sox6*** **rescues** ***tnnc1b*** **and** ***smyhc1*** **expression in** ***prdm1a*** ^***nrd***^ **mutants. (A-D)** Cross sections through the trunk region of embryos that were hybridized with the *smyhc1* probe at 30 hours postfertilization (hpf): note that migration of the slow-twitch progenitors is disrupted in *prdm1a* mutants, and the expression of *smyhc1* is dramatically downregulated (cf **A** and **C**); in the *prdm1a;sox6* double mutant, *smyhc1* expression is restored in the adaxial cells, though their migration still appears disrupted (cf. **C** and **D**). **(E-H)** Similar cross sections of wild-type and mutant embryos hybridized with the *tnnc1b* probe. Expression of *tnnc1b* is completely lost *prdm1a* mutants (cf **E** and **G**); expression is restored throughout the myotomes in the *prdm1a;sox6* double mutants, though at lower levels than in the *sox6* single mutant (cf. **F** and **H**) are slow specific genes, which are expressed in the superficial slow-twitch fibers in wild-type embryos at 30 hpf **(A and B)**.

Loss of Prdm1a function causes ectopic expression of *sox6* in adaxial cells and concomitantly the loss or strong downregulation of slow-twitch muscle gene expression [13,21,22]; (Figure 6C and G). In the *sox6;prdm1*a double homozygotes, expression of *smyhc1* in adaxial cells was partially restored (Figure 6D), while *tnnc1b* was uniformly expressed throughout the myotome (Figure 6H). The migration of the slow-twitch progenitors is also disrupted in *prdm1a* mutants (Elworthy *et al*. [22]); this effect was not rescued in *prdm1a;sox6* double mutants (YO and PWI, unpublished observations: see also Figure 6D).

### Loss of Sox6 results in altered physiology of fast fibers

**Figure 7:**
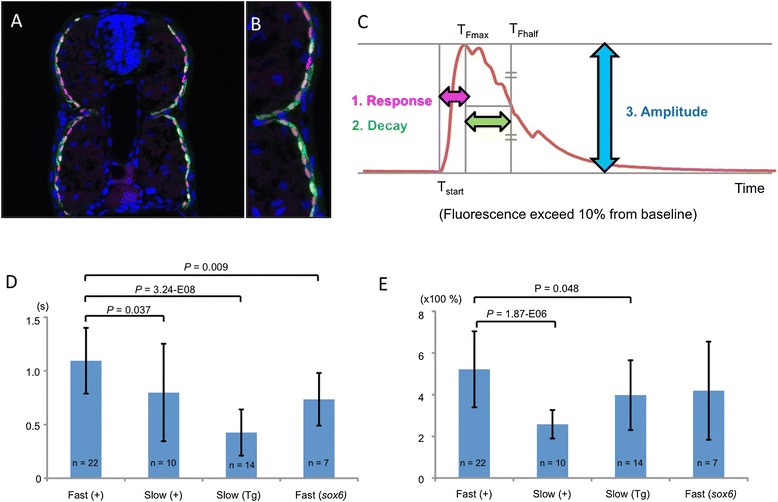
**Sox6 controls the Ca2+ response of fast-twitch fibers. (A,B)** Cross section through a trunk somite of a 48 hours postfertilization (hpf) Tg(*smyhc1:GCaMP3)* ^*i280*^ embryo, showing expression of GCaMP3 (green) is restricted to slow-twitch fibers, revealed by mAbF59 labeling (red). Nuclei are stained with DAPI (blue). **(C)** Stereotypical representation of the GCaMP3 signal in response to chemical stimulation indicating the three parameters measured in this analysis. **(D)** Response time in seconds (s) and **(E)** amplitude of Ca2+ response in fast and slow-twitch fibers of Tg(*smyhc1:GCaMP3)* ^*i280*^ (Tg) and transient transgenic wild-type (+) and *sox6* mutant larvae (52 to 55 hpf); Student’s two-tailed, unpaired t-test; Error bars represent standard deviation.

Prompted by the ectopic expression of the slow-twitch specific ryanodine receptor encoded by *ryr1a* in fast-twitch fibers in *sox6* mutant larvae, we analyzed Ca2+ flux in muscle fibers, using transgenes driving muscle specific expression of the fluorescent calcium sensor GCaMP3 [38,39]. Animals carrying the *smyhc1:GCaMP3* transgene express the sensor specifically in superficial slow-twitch muscle fibers (Figure 7A and B) while the *mylz2:GCaMP* transgene is specifically expressed in fast-twitch fibers [34]. Pentylenetetrazole (PTZ) treatment of transgenic larvae induced a rapid increase in fluorescence intensity followed by a return to the baseline level, reflecting Ca2+ flux from the sarcoplasmic reticulum into the muscle fiber. We analyzed three components of this calcium response: 1) time to maximum fluorescence intensity, 2) decay time to 50% of maximum fluorescence intensity, and 3) fluorescence amplitude (Figure 7C).

In wild-type larvae, the response time of slow-twitch fibers was shorter than in fast-twitch fibers, while the change in amplitude was smaller (Figure 7D and E). Notably, the response time of *sox6* mutant fast-twitch fibers was significantly shorter than in their wild-type counterparts, resembling more that of wild-type slow-twitch fibers, whereas the amplitude change was unaffected. The fluorescence decay time showed no significant difference between slow-twitch and fast-twitch fibers in both wild-type and *sox6* mutant fibers, indicating that the recovery mechanism of calcium ion flux is similar in both types of fiber (data not shown).

### Loss of Sox6 disrupts muscle fiber type at adult stages

**Figure 8:**
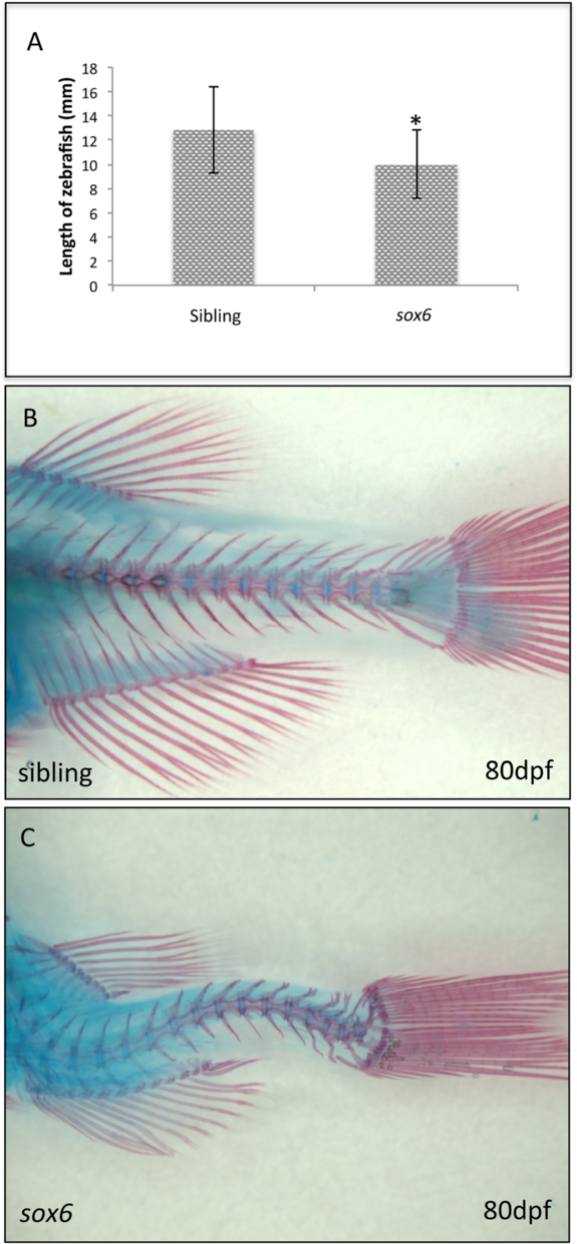
***sox6*** **mutant adults are small and have spinal deformities. (A)** *sox6* mutant fish are significantly smaller than siblings by 1 month. Student’s two-tailed, unpaired t-test *P* <0.05, error bars represent standard deviation, sibling n = 17, mutant n = 17, asterisk indicates significance. **(B and C)** By 80 dpf, there is clear curvature of the spine in all surviving homozygous *sox6* mutant fish **(C)** as compared to siblings **(B)**.

While homozygous *sox6* mutant embryos and larvae displayed no observable morphological phenotype, by one month of age they were significantly smaller than their siblings (Figure 8A) and exhibited a slight curvature of the spine, a phenotype that became more severe with age. The extent of the curvature varied between individuals: some fish exhibited only a slight kink, while others displayed a massive crumpling of the spine. By 80 dpf, all surviving homozygous *sox6* mutants displayed some degree of curvature as revealed by alizarin red staining (Figure 8B and C).

**Figure 9:**
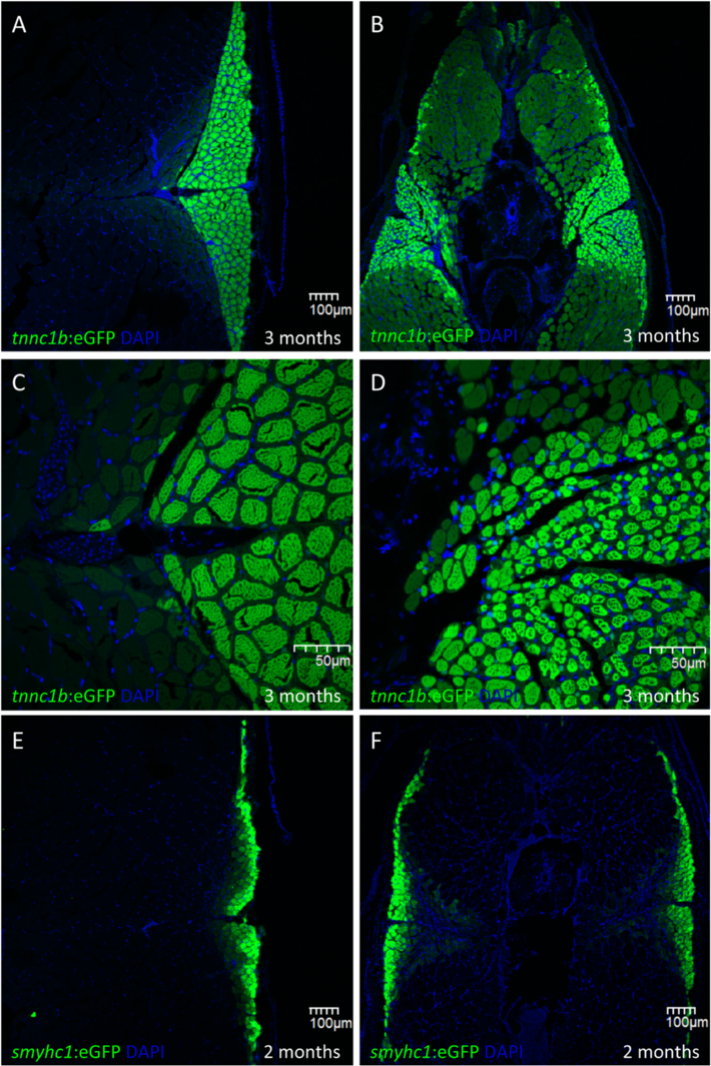
**Disruption of fiber type identity in adult** ***sox6*** **mutant fish. (A,B)** at 3 months, expression of the *tnnc1b:eGFP* reporter is restricted to the medio-lateral wedge of slow-twitch fibers in wild-type fish. **(C,D)** In *sox6* mutants, the reporter gene is also expressed at lower levels in fast-twitch fibers while the slow-twitch fibers extend closer to the midline. The diameter of the slow-twitch fibers is significantly reduced compared to wild-type (cf. **B** and **D**). **(E)** The *smyhc1:GFP* reporter is expressed at high levels in the slow-twitch fiber of 2-month-old fish. **(F)** the reporter shows a similar pattern of expression in *sox6* mutants at the same stage, with lower level expression extending more medially than in wild type.

Around 10% of homozygotes could survive to adulthood if raised in isolation from their siblings; these failed to breed, most likely due to the morphological abnormalities. Adult muscle fibers were examined by visualizing the expression of the *tnnc1b*:eGFP and *smyhc1:eGFP* transgenes in cryostat sections through the trunk regions of *sox6* mutants and their siblings. This revealed that the ectopic expression of *tnnc1b* seen in mutant embryos and larvae persists into adulthood (Figure 9; cf. A and B). The level of expression of *tnnc1b:eGFP* in the fast domain, however, was weaker than in the slow-twitch domain, resembling more that of intermediate fibers in wild type. Moreover, the morphology of the mutant myotome was altered compared to that of wild-type siblings, varying in shape and size between individuals. The wedge of eGFP-positive slow-twitch muscle, known as the *lateralis superficialis* [7,22] extended more medially towards the midline of *sox6* mutants (Figure 9; cf. A and B). In addition, the slow-twitch fibers were significantly smaller than in wild-type fish (Figure 9; cf. C and D).

By contrast, expression of *smyhc1:eGFP* remained restricted to the slow-twitch fibers in *sox6* mutants. As in mutant embryos, sporadic ectopic expression of the *smyhc1* reporter was observed in only a small number of fast-twitch fibers (data not shown). There was a slight medial expansion of the eGFP-positive domain, with some cells in the most medially located muscle fibers faintly expressing eGFP, as well as a dorsal and ventral expansion in the expression domain (Figure 9E and F).

## Discussion

Previous studies have implicated the transcription factor Sox6 in the control of skeletal muscle fiber type identity both in mammals and fish [17-19,21]. In *Sox6* mutant mice, various fast specific genes are downregulated in fast-twitch fibers while a number of slow fiber specific genes are upregulated; these include several myosin heavy chain genes as well as all three of the genes encoding the slow troponin subunits *Tnnc1,Tnni1 and Tnnt1* [17-19]. By contrast, we observed only limited ectopic expression of *smyhc1* at 30 hpf, with *mylz10* and *tmp2* also showing limited ectopic expression in the fast fibers by 3 dpf*.*

On the other hand, ectopic Sox6 activity was sufficient to reduce expression all of these slow-twitch specific genes in the adaxial cells and consistent with this, we found that mutation of the putative Sox binding sites in the *smyhc1* cis regulatory fragment resulted in ectopic expression of a GFP reporter gene in fast-twitch fibers.

One possible explanation for these paradoxical findings could be the presence of a paralogous *sox6* gene in the zebrafish genome, a consequence of the additional round of genome duplication that has occurred in teleosts [40]. Indeed, such teleost-specific *sox6* paralogs were first described in puffer fish [41] and subsequent genome sequence analyses have revealed similar *sox6* duplicates in Medaka and stickleback. Surprisingly, however, the zebrafish genome lacks the *sox6a* locus found in other teleosts (Additional file 7: Figure S7), thus ruling out this redundancy hypothesis.

Another potential explanation could be a partial functional overlap between Sox6 and the closely related Sox5 protein; indeed it is well established that Sox5 and Sox6 function redundantly in a number of contexts in mammals [42-44]. To address this possibility, we generated *sox5* null alleles using zinc finger nucleases (SE and PWI, unpublished data) and made compound homozygotes; however, we found no evidence of de-repression of additional slow-twitch genes in the double mutants (Additional file 8: Figure S8).

Nevertheless, the fact that ectopic Sox6 can repress expression of all of the slow-twitch genes we have assayed implies that some other Sox protein may act in parallel to Sox6 to regulate fiber type identity. There is as yet, however, no obvious candidate for such an additional Sox family member involved in myogenesis. A further puzzle is presented by our analysis of the *tnnc1b* promoter; this identified an upstream fragment sufficient to drive reporter gene expression specifically in slow-twitch fibers, which like the *smyhc1* upstream regulatory element described previously, contains multiple putative Sox6 binding sites. Surprisingly, however, mutation of these sites failed to cause ectopic reporter gene expression in fast-twitch fibers. Moreover, partial deletion of this fragment identified a minimal upstream element devoid of canonical Sox6 binding sites that retains slow-specific enhancer activity. It is of course possible that other cryptic Sox6 binding sites are present in this fragment or that Sox6 binds via interaction with another DNA binding protein. In the absence of a Sox6 antibody that can be used in chromatin precipitation assays, we have been unable to address the interaction between Sox6 and this element directly.

The homeodomain transcription factors Six1a and Pbx have previously been implicated in the transcriptional activation of fast-specific genes in zebrafish [45,46]. Since both proteins can also act as transcriptional repressors, it seems possible that they might act in concert with Sox6 to repress the expression of slow-specific genes in the fast-twitch fibers muscle. Morpholino-mediated knockdown of either gene in *sox6* mutant embryos, however, had no discernible effect on the expression of the *smyhc1:gfp* transgene (data not shown).

In any case, it is clear from our analysis that Sox6 is not the sole mediator of slow-twitch gene repression in fast-twitch fibers. In this respect, it is worth noting that neither *Ppargc1a* (PGC-1a) nor *Sdha*, both of which are associated with the oxidative metabolism characteristic of slow-twitch fibers, are upregulated in fast-twitch fibers of mouse *Sox6* mutants [19,20]. Nevertheless, the disparity between mammals and zebrafish in the selective repression of sarcomeric protein genes in the latter but not the former is striking and worthy of further investigation.

Using fiber type-specific expression of the Ca2+ reporter GCaMP3, we found that fast- and slow-twitch fibers show different calcium responses and that loss of Sox6 function modified the fast-twitch fiber specific response. In a previous study, the calcium indicator Calcium Green-1 dextran was used to analyze Ca2+ transients in wild-type and mutant larvae [32]. The responses reported for slow and fast-twitch muscles in wild-type embryos appeared quite similar, although no statistical analysis was performed to confirm this. The discrepancy between these earlier results and our findings could possibly be ascribed to the different mode of muscle stimulation - mechanosensory stimulation versus drug-induced convulsion - used in the two studies. Nevertheless, it is notable that loss of Sox6 function modified the response of the fast-twitch fibers in our analysis. We did not, however, observe a significant change in the half decay time of the Ca2+ signal in *sox6* mutant embryos. On the other hand, it was reported that decay time is delayed in the *atp2a1* mutant that encodes ATPase Ca2+ pump SERCA1 [47]. This suggests that expression of SERCA1 is independent of Sox6 function.

In contrast to the perinatal lethality of the mouse *Sox6* mutation, a small proportion (approximately 10%) of zebrafish homozygous for the *sox6* null allele survived to adulthood. These fish showed persistent ectopic expression of the *tnnc1b* gene throughout the fast-twitch fibers indicating a continuous requirement for Sox6 in the maintenance of fiber type identity. In addition, the muscle blocks were misshapen and reduced in size, while slow-twitch fibers appeared significantly smaller than in wild type. The mutant fish also exhibited a marked scoliosis; while this may be a secondary consequence of the defects in the skeletal muscle, it could also reflect a direct requirement for Sox6 in the vertebral column. In this regard, it is notable that previous studies in mouse have revealed a role for both Sox5 and Sox6 in the formation of the extracellular matrix sheath of the notochord and of the nucleus pulposus, the gelatinous central portion of the intervertebral discs [48]. Further analysis is required to determine whether the scoliosis in *sox6* mutant fish is a reflection of such a requirement.

## Conclusions

Our analysis has established that the Sox6 transcription factor is a key regulator of fast-twitch muscle fiber differentiation in the zebrafish, a role similar to that ascribed to its murine ortholog. In the absence of Sox6 function, genes encoding slow-twitch specific sarcomeric proteins are ectopically expressed in fast-twitch fibers, the physiological properties of which are correspondingly shifted towards those of slow-twitch fibers. Not all slow-twitch specific genes are de-repressed in the absence of Sox6 function, however, which implies that additional factors act to suppress the slow-twitch differentiation program in fast-twitch fibers.

## Additional files

Additional file 1: Figure S1. The expression of slow-specific genes is downregulated in *actin:GAL4; UAS:sox6-GFP* embryos. The slow-twitch specific expression of *tnnt1* (A), *tnni1a* (C), *mylz10* (E), *tpm2* (G) and *ryr1a* (I) is downregulated in slow muscle fibers ectopically expressing Sox6-GFP (B,D,F,H,J). Additional file 2: Figure S2. Misexpression of Sox6 has no effect on the expression of *tnnc1b* in the head muscles. (A-C ) *tnnc1b* expression in the head muscles of 3 dpf wild type embryos. (D-F) *tnnc1b* expression in embryos expressing Sox6-GFP in head muscles. There appears to be no difference in the expression of *tnnc1b* between wild type and *actin:GAL4:UAS:sox6-GFP* fish. Additional file 3: Figure S3. GFP expression is unaffected by mutation of potential Sox6 binding sites in the +2.5kb + intron1 *tnnc1b:eGFP* reporter. Expression remains restricted to the slow-twitch domain following mutation of one or more putative Sox6 binding sites (A-F). Additional file 4: Figure S4.
*In situ* hybridization for *tnnc1b* mRNA reveals no difference in expression between wild type and *sox6* mutants in the craniofacial muscles. Additional file 5: Figure S5. Slow-specific muscle genes show a variable response to mutation of *sox6*. Like *tnnc1b* and *ryr1a, tnnt1* is ectopically expressed throughout the fast domain of *sox6* homozygous mutant embryos at 30 hpf (A and F). Conversely *prox1a* expression is unaffected in *sox6* mutant embryos at 22 hpf when transcript levels can be detected (B and G). Expression of *tnni1a* (C and H), *mylz10* (D and I) and *tpm2* (E and J) is unaffected at 30 hpf in *sox6* mutants. Additional file 6: Figure S6. Expression of fast specific troponin subunit genes in wild type and *sox6* mutant embryos. Additional file 7: Figure S7. Chromosomal location of *sox6* genes in different fish species and *H. sapiens*. Two *sox6* paralogs are present in the genomes of Fugu, stickleback and Medaka. The *sox6B* locus in each of these species is located in a chromosomal region that displays conserved synteny with the locations of the zebrafish and human *sox6* gene. The *sox6A* genes of Fugu and stickleback are also located in a region of conserved synteny. This conservation has been lost in zebrafish along with the *sox6A* paralog (indicated by “?”). Additional file 8: Figure S8. Expression of *smyhc1, myh7b* and *tnnc1b* in wild type, *sox5, sox6* and *sox5; sox6* double mutants.

## Abbreviations

dpf: days postfertilization
Hh: hedgehog
hpf: hours postfertilization
MPs: muscle pioneers
MRFs: myogenic regulatory factors
PTZ: pentylenetetrazole
SSF: superficial slow-twitch fibers
ZFN: zinc-finger nucleases

## References

[1] Schiaffino S, Reggiani C (2011). Fiber types in mammalian skeletal muscles. Physiol Rev.

[2] Jackson HE, Ingham PW (2013). Control of muscle fibre-type diversity during embryonic development: the zebrafish paradigm. Mech Dev.

[3] Weinberg ES, Allende ML, Kelly CS, Abdelhamid A, Murakami T, Andermann P, Doerre OG, Grunwald DJ, Riggleman B (1996). Developmental regulation of zebrafish MyoD in wild-type, no tail and spadetail embryos. Development.

[4] Blagden CS, Currie PD, Ingham PW, Hughes SM (1997). Notochord induction of zebrafish slow muscle mediated by Sonic hedgehog. Genes Dev.

[5] Coutelle O, Blagden CS, Hampson R, Halai C, Rigby PW, Hughes SM (2001). Hedgehog signalling is required for maintenance of myf5 and myoD expression and timely terminal differentiation in zebrafish adaxial myogenesis. Dev Biol.

[6] Hammond CL, Hinits Y, Osborn DP, Minchin JE, Tettamanti G, Hughes SM (2007). Signals and myogenic regulatory factors restrict pax3 and pax7 expression to dermomyotome-like tissue in zebrafish. Dev Biol.

[7] Devoto SH, Melançon E, Eisen JS, Westerfield M (1996). Identification of separate slow and fast muscle precursor cells in vivo, prior to somite formation. Development.

[8] Barresi MJ, Stickney HL, Devoto SH (2000). The zebrafish slow-muscle-omitted gene product is required for Hedgehog signal transduction and the development of slow muscle identity. Development.

[9] Baxendale S, Davison C, Muxworthy C, Wolff C, Ingham PW, Roy S (2004). The B-cell maturation factor Blimp-1 specifies vertebrate slow-twitch muscle fiber identity in response to Hedgehog signaling. Nat Genet.

[10] Du SJ, Devoto SH, Westerfield M, Moon RT (1997). Positive and negative regulation of muscle cell identity by members of the hedgehog and TGF-beta gene families. J Cell Biol.

[11] Hirsinger E, Stellabotte F, Devoto SH, Westerfield M (2004). Hedgehog signaling is required for commitment but not initial induction of slow muscle precursors. Dev Biol.

[12] Lewis KE, Currie PD, Roy S, Schauerte H, Haffter P, Ingham PW (1999). Control of muscle cell-type specification in the zebrafish embryo by Hedgehog signalling. Dev Biol.

[13] Roy S, Wolff C, Ingham PW (2001). The u-boot mutation identifies a Hedgehog-regulated myogenic switch for fiber-type diversification in the zebrafish embryo. Genes Dev.

[14] Felsenfeld AL, Curry M, Kimmel CB (1991). The fub-1 mutation blocks initial myofibril formation in zebrafish muscle pioneer cells. Dev Biol.

[15] Hatta K, Bremiller R, Westerfield M, Kimmel CB (1991). Diversity of expression of engrailed-like antigens in zebrafish. Development.

[16] Henry CA, Amacher SL (2004). Zebrafish slow muscle cell migration induces a wave of fast muscle morphogenesis. Dev Cell.

[17] Hagiwara N, Ma B, Ly A (2005). Slow and fast fiber isoform gene expression is systematically altered in skeletal muscle of the Sox6 mutant, p100H. Dev Dyn.

[18] Hagiwara N, Yeh M, Liu A (2007). Sox6 is required for normal fiber type differentiation of fetal skeletal muscle in mice. Dev Dyn.

[19] An CI, Dong Y, Hagiwara N (2011). Genome-wide mapping of Sox6 binding sites in skeletal muscle reveals both direct and indirect regulation of muscle terminal differentiation by Sox6. BMC Dev Biol.

[20] Quiat D, Voelker KA, Pei J, Grishin NV, Grange RW, Bassel-Duby R, Olson EN (2011). Concerted regulation of myofiber-specific gene expression and muscle performance by the transcriptional repressor Sox6. Proc Natl Acad Sci U S A.

[21] von Hofsten J, Elworthy S, Gilchrist MJ, Smith JC, Wardle FC, Ingham PW (2008). Prdm1- and Sox6-mediated transcriptional repression specifies muscle fibre type in the zebrafish embryo. EMBO Rep.

[22] Elworthy S, Hargrave M, Knight R, Mebus K, Ingham PW (2008). Expression of multiple slow myosin heavy chain genes reveals a diversity of zebrafish slow twitch muscle fibres with differing requirements for Hedgehog and Prdm1 activity. Development.

[23] Hernandez-Lagunas L, Choi IF, Kaji T, Simpson P, Hershey C, Zhou Y, Zon L, Mercola M, Artinger KB (2005). Zebrafish narrowminded disrupts the transcription factor prdm1 and is required for neural crest and sensory neuron specification. Dev Biol.

[24] Maurya AK, Tan H, Souren M, Wang X, Wittbrodt J, Ingham PW (2011). Integration of Hedgehog and BMP signalling by the engrailed2a gene in the zebrafish myotome. Development.

[25] Lee EC, Yu D, Martinez de Velasco J, Tessarollo L, Swing DA, Court DL, Jenkins NA, Copeland NG (2001). A highly efficient Escherichia coli-based chromosome engineering system adapted for recombinogenic targeting and subcloning of BAC DNA. Genomics.

[26] Kawakami K (2004). Transgenesis and gene trap methods in zebrafish by using the Tol2 transposable element. Methods Cell Biol.

[27] Balciunas D, Wangensteen KJ, Wilber A, Bell J, Geurts A, Sivasubbu S, Wang X, Hackett PB, Largaespada DA, McIvor RS, Ekker SC (2006). Harnessing a high cargo-capacity transposon for genetic applications in vertebrates. PLoS Genet.

[28] MacDonald RB, Debiais-Thibaud M, Talbot JC, Ekker M (2010). The relationship between dlx and gad1 expression indicates highly conserved genetic pathways in the zebrafish forebrain. Dev Dyn.

[29] Braissant O, Wahli W (1998). Differential expression of peroxisome proliferator-activated receptor-alpha, −beta, and -gamma during rat embryonic development. Endocrinology.

[30] Oxtoby E, Jowett T (1993). Cloning of the zebrafish krox-20 gene (krx-20) and its expression during hindbrain development. Nucleic Acids Res.

[31] Wang X, Ono Y, Tan SC, Chai RJ, Parkin C, Ingham PW (2011). Prdm1a and miR-499 act sequentially to restrict Sox6 activity to the fast-twitch muscle lineage in the zebrafish embryo. Development.

[32] Hirata H, Watanabe T, Hatakeyama J, Sprague SM, Saint-Amant L, Nagashima A, Cui WW, Zhou W, Kuwada JY (2007). Zebrafish relatively relaxed mutants have a ryanodine receptor defect, show slow swimming and provide a model of multi-minicore disease. Development.

[33] Glasgow E, Tomarev SI (1998). Restricted expression of the homeobox gene prox 1 in developing zebrafish. Mech Dev.

[34] Baxendale S, Holdsworth CJ, Meza Santoscoy PL, Harrison MR, Fox J, Parkin CA, Ingham PW, Cunliffe VT (2012). Identification of compounds with anti-convulsant properties in a zebrafish model of epileptic seizures. Dis Model Mech.

[35] Yasmin L, Kinoshita S, Asaduzzaman M, Akolkar DB, Ikeda D, Ono Y, Watabe S (2011). A 5’-flanking region of embryonic-type myosin heavy chain gene, MYH(M)(7)(4)(3)(−)(2), from torafugu Takifugu rubripes regulates developmental muscle-specific expression. Comp Biochem Physiol Part D Genomics Proteomics.

[36] Parmacek MS, Ip HS, Jung F, Shen T, Martin JF, Vora AJ, Olson EN, Leiden JM (1994). A novel myogenic regulatory circuit controls slow/cardiac troponin C gene transcription in skeletal muscle. Mol Cell Biol.

[37] Meng X, Noyes MB, Zhu LJ, Lawson ND, Wolfe SA (2008). Targeted gene inactivation in zebrafish using engineered zinc-finger nucleases. Nat Biotechnol.

[38] Nakai J, Ohkura M, Imoto K (2001). A high signal-to-noise Ca(2+) probe composed of a single green fluorescent protein. Nat Biotechnol.

[39] Tian L, Hires SA, Mao T, Huber D, Chiappe ME, Chalasani SH, Petreanu L, Akerboom J, McKinney SA, Schreiter ER, Bargmann CI, Jayaraman V, Svoboda K, Looger L (2009). Imaging neural activity in worms, flies and mice with improved GCaMP calcium indicators. Nat Methods.

[40] Amores A, Force A, Yan YL, Joly L, Amemiya C, Fritz A, Ho RK, Langeland J, Prince V, Wang YL, Westerfield M, Ekker M, Postlethwait JH (1998). Zebrafish hox clusters and vertebrate genome evolution. Science.

[41] Koopman P, Schepers G, Brenner S, Venkatesh B (2004). Origin and diversity of the SOX transcription factor gene family: genome-wide analysis in Fugu rubripes. Gene.

[42] Han Y, Lefebvre V (2008). L-Sox5 and Sox6 drive expression of the aggrecan gene in cartilage by securing binding of Sox9 to a far-upstream enhancer. Mol Cell Biol.

[43] Lefebvre V, Li P, de Crombrugghe B (1998). A new long form of Sox5 (L-Sox5), Sox6 and Sox9 are coexpressed in chondrogenesis and cooperatively activate the type II collagen gene. EMBO J.

[44] Smits P, Li P, Mandel J, Zhang Z, Deng JM, Behringer RR, de Crombrugghe B, Lefebvre V (2001). The transcription factors L-Sox5 and Sox6 are essential for cartilage formation. Dev Cell.

[45] Bessarab DA, Chong SW, Srinivas BP, Korzh V (2008). Six1a is required for the onset of fast muscle differentiation in zebrafish. Dev Biol.

[46] Maves L, Waskiewicz AJ, Paul B, Cao Y, Tyler A, Moens CB, Tapscott SJ (2007). Pbx homeodomain proteins direct Myod activity to promote fast-muscle differentiation. Development.

[47] Hirata H, Saint-Amant L, Waterbury J, Cui W, Zhou W, Li Q, Goldman D, Granato M, Kuwada JY (2004). Accordion, a zebrafish behavioral mutant, has a muscle relaxation defect due to a mutation in the ATPase Ca2+ pump SERCA1. Development.

[48] Smits P, Lefebvre V (2003). Sox5 and Sox6 are required for notochord extracellular matrix sheath formation, notochord cell survival and development of the nucleus pulposus of intervertebral discs. Development.

