# Supplementary figures and images for: The role of Sox6 in zebrafish muscle fiber type specification
Source: Skelet Muscle. 2015 Jan 27;5(1):2. doi: 10.1186/s13395-014-0026-2 (PMC4323260; doi:10.1186/s13395-014-0026-2)

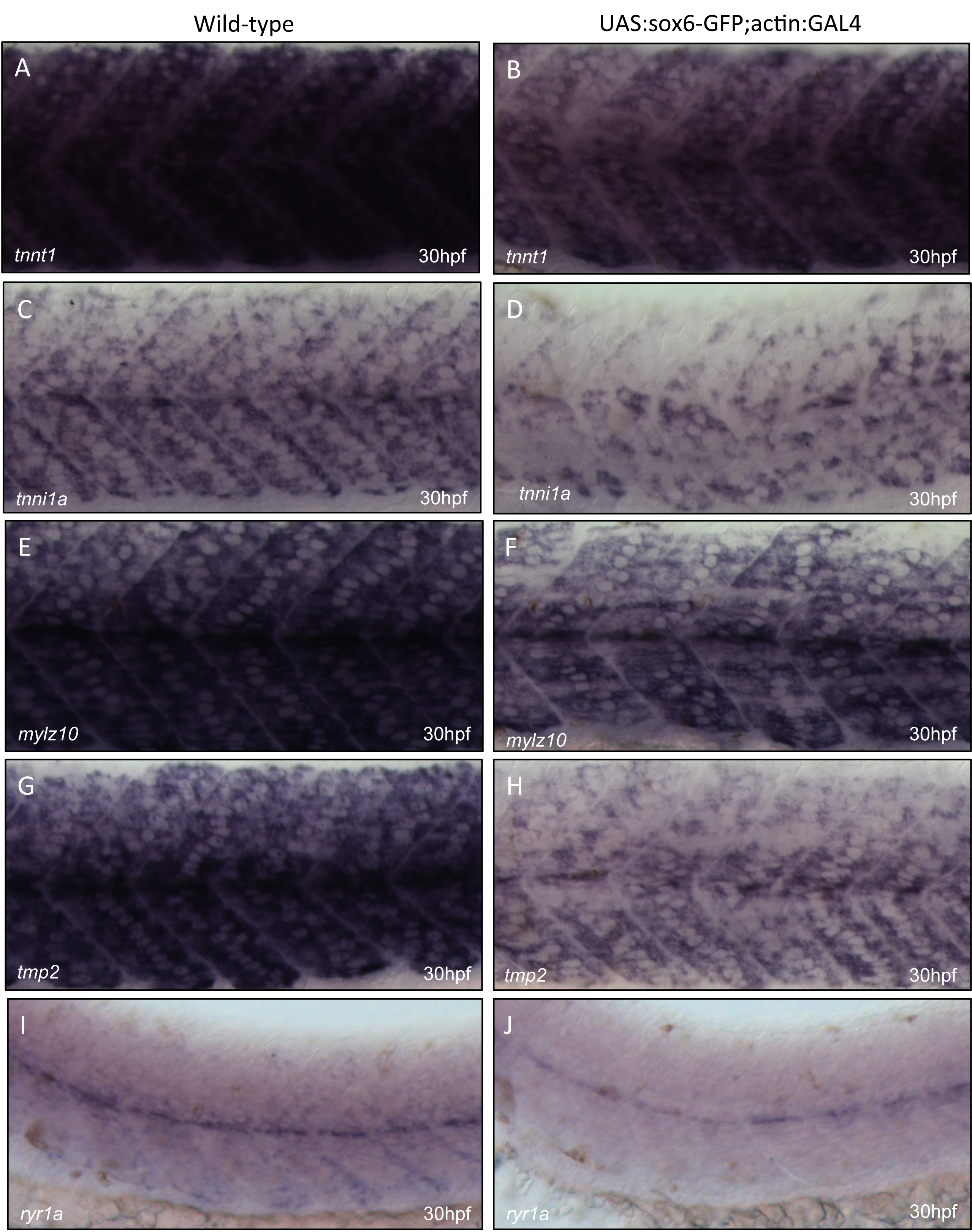

Supplement: Additional file 1: Figure S1. — The expression of slow-specific genes is downregulated in actin:GAL4; UAS:sox6-GFP embryos. The slow-twitch specific expression of tnnt1 (A), tnni1a (C), mylz10 (E), tpm2 (G) and ryr1a (I) is downregulated in slow muscle fibers ectopically expressing Sox6-GFP (B,D,F,H,J). [file 13395_2014_26_MOESM1_ESM.jpeg]

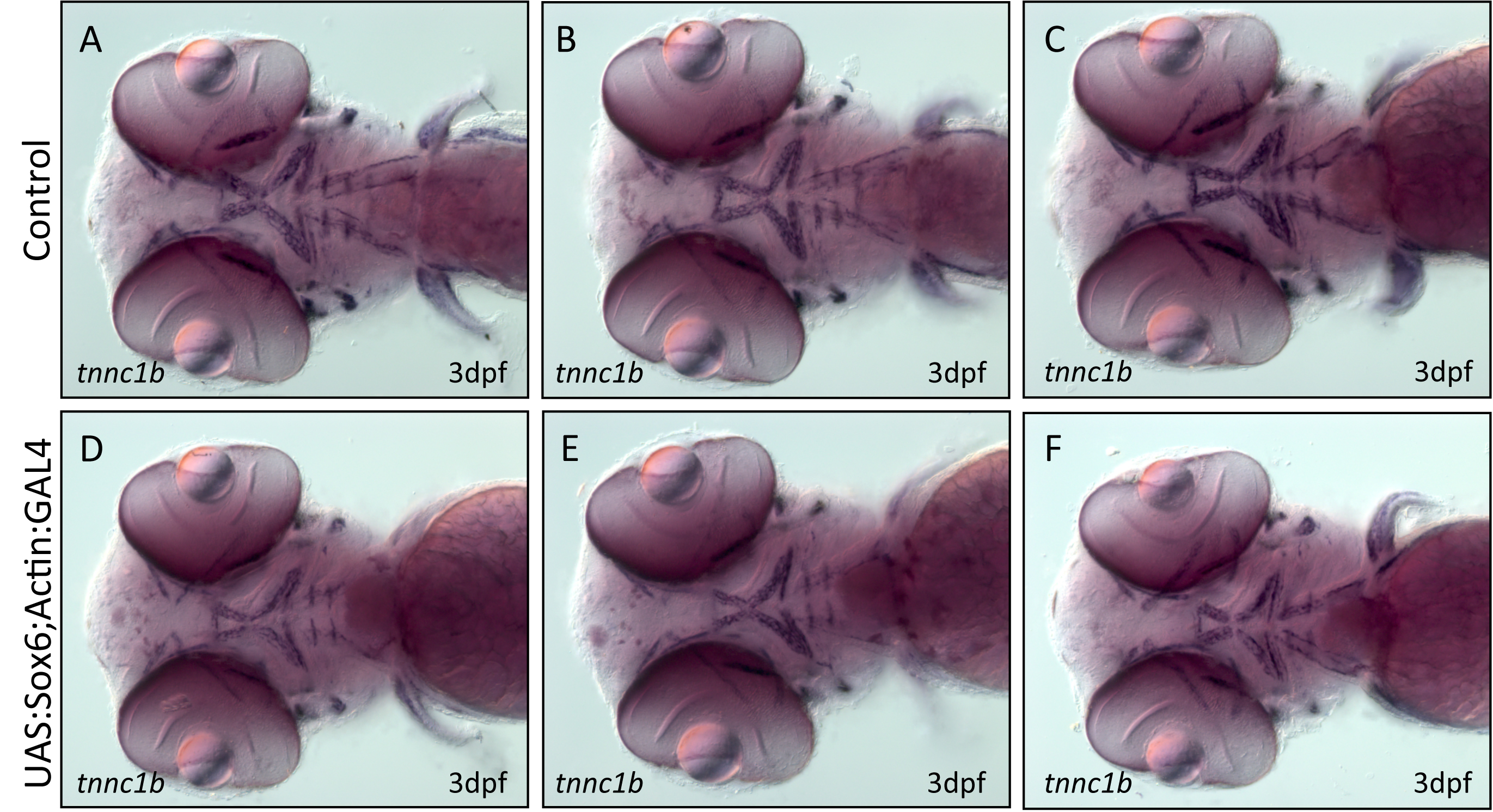

Supplement: Additional file 2: Figure S2. — Misexpression of Sox6 has no effect on the expression of tnnc1b in the head muscles. (A-C ) tnnc1b expression in the head muscles of 3 dpf wild type embryos. (D-F) tnnc1b expression in embryos expressing Sox6-GFP in head muscles. There appears to be no difference in the expression of tnnc1b between wild type and actin:GAL4:UAS:sox6-GFP fish. [file 13395_2014_26_MOESM2_ESM.jpeg]

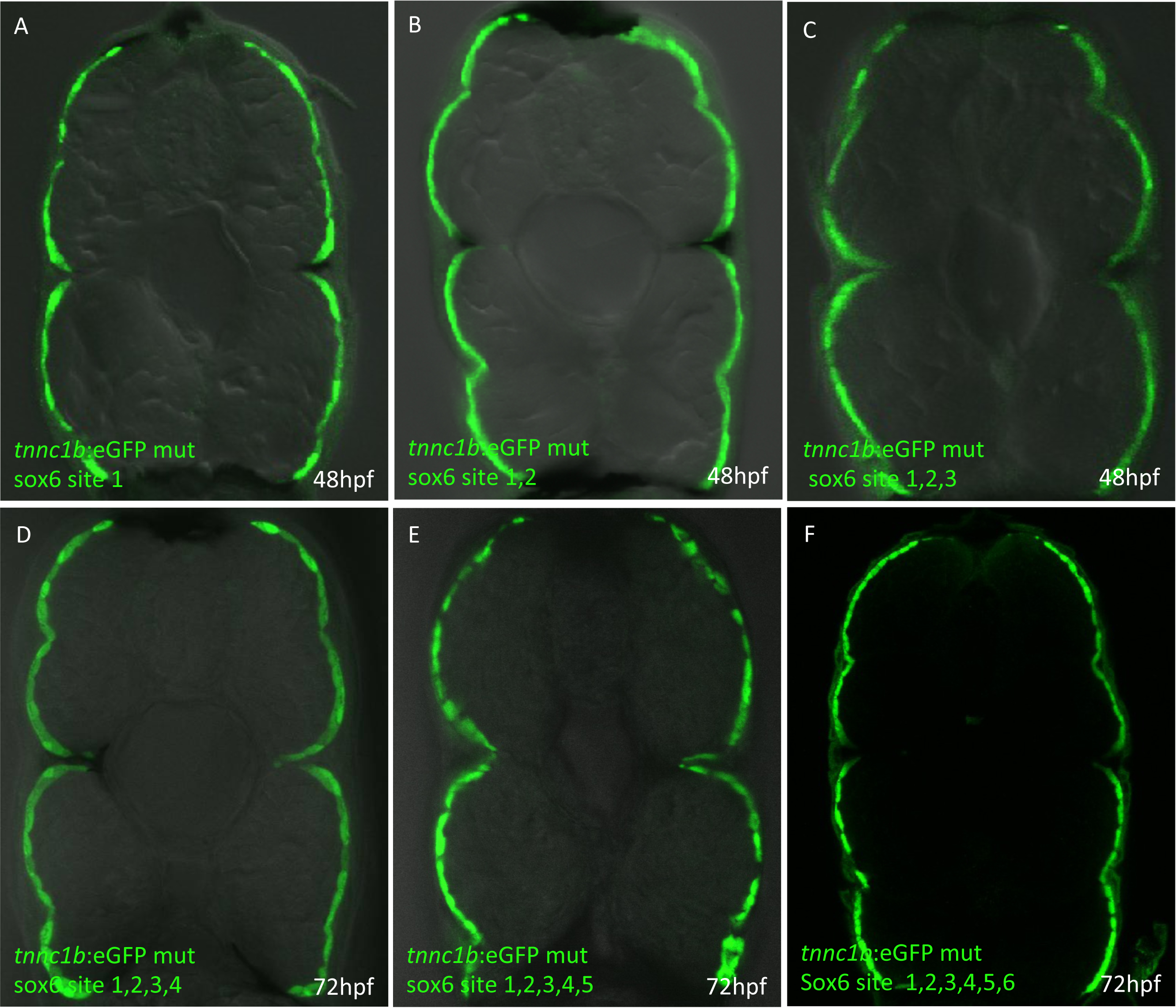

Supplement: Additional file 3: Figure S3. — GFP expression is unaffected by mutation of potential Sox6 binding sites in the +2.5kb + intron1 tnnc1b:eGFP reporter. Expression remains restricted to the slow-twitch domain following mutation of one or more putative Sox6 binding sites (A-F). [file 13395_2014_26_MOESM3_ESM.jpeg]

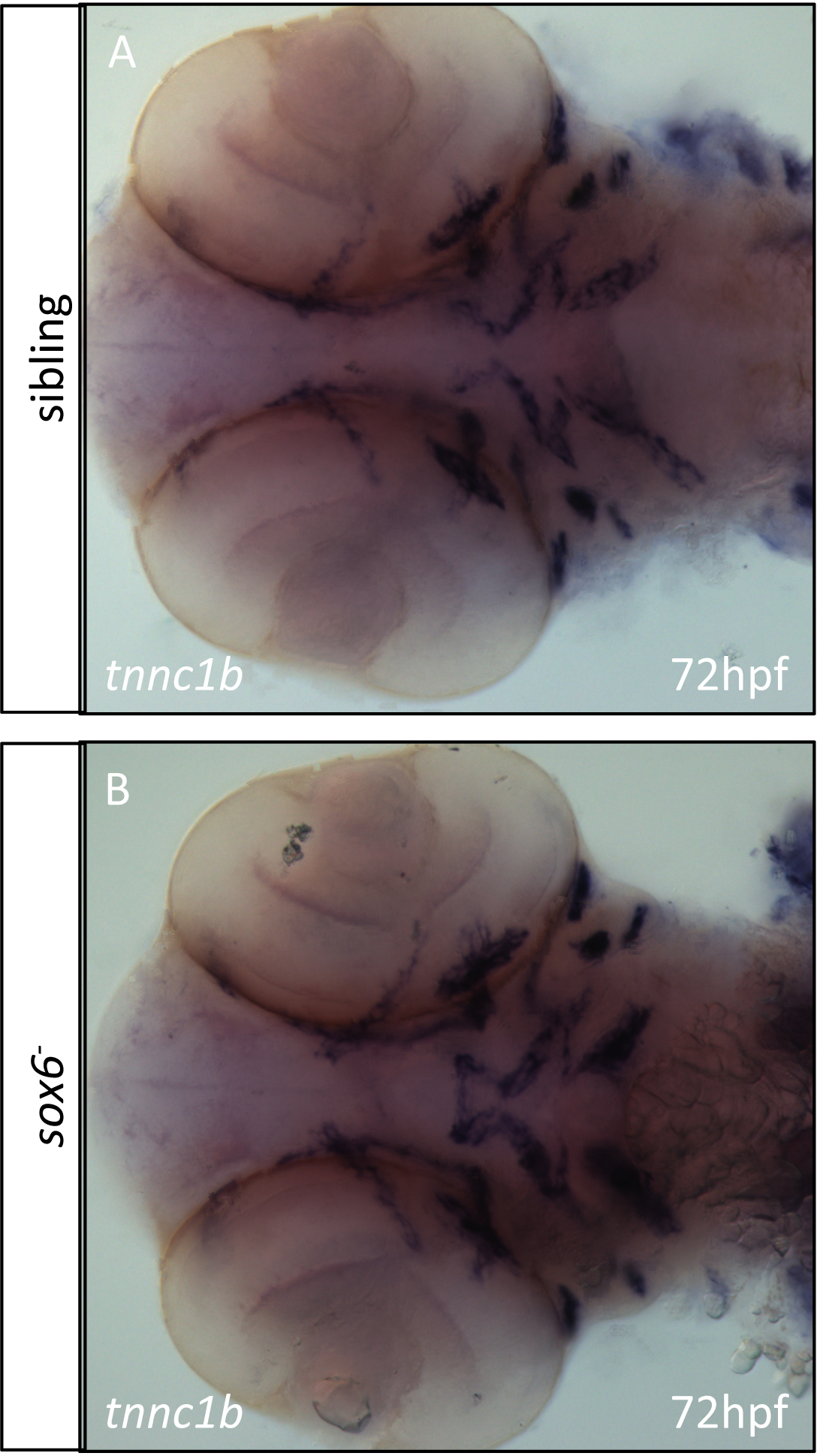

Supplement: Additional file 4: Figure S4. — In situ hybridization for tnnc1b mRNA reveals no difference in expression between wild type and sox6 mutants in the craniofacial muscles. [file 13395_2014_26_MOESM4_ESM.jpeg]

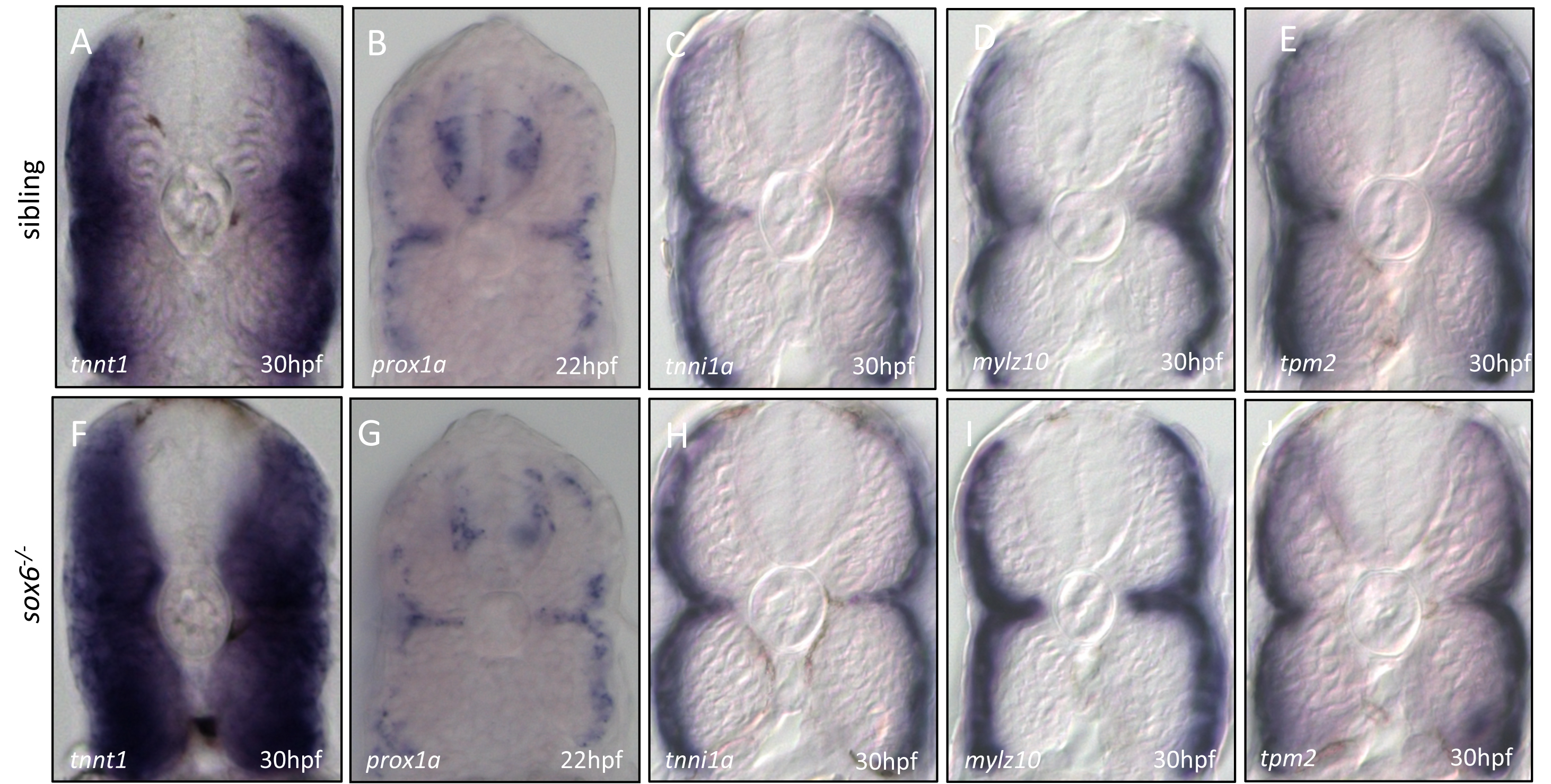

Supplement: Additional file 5: Figure S5. — Slow-specific muscle genes show a variable response to mutation of sox6. Like tnnc1b and ryr1a, tnnt1 is ectopically expressed throughout the fast domain of sox6 homozygous mutant embryos at 30 hpf (A and F). Conversely prox1a expression is unaffected in sox6 mutant embryos at 22 hpf when transcript levels can be detected (B and G). Expression of tnni1a (C and H), mylz10 (D and I) and tpm2 (E and J) is unaffected at 30 hpf in sox6 mutants. [file 13395_2014_26_MOESM5_ESM.jpeg]

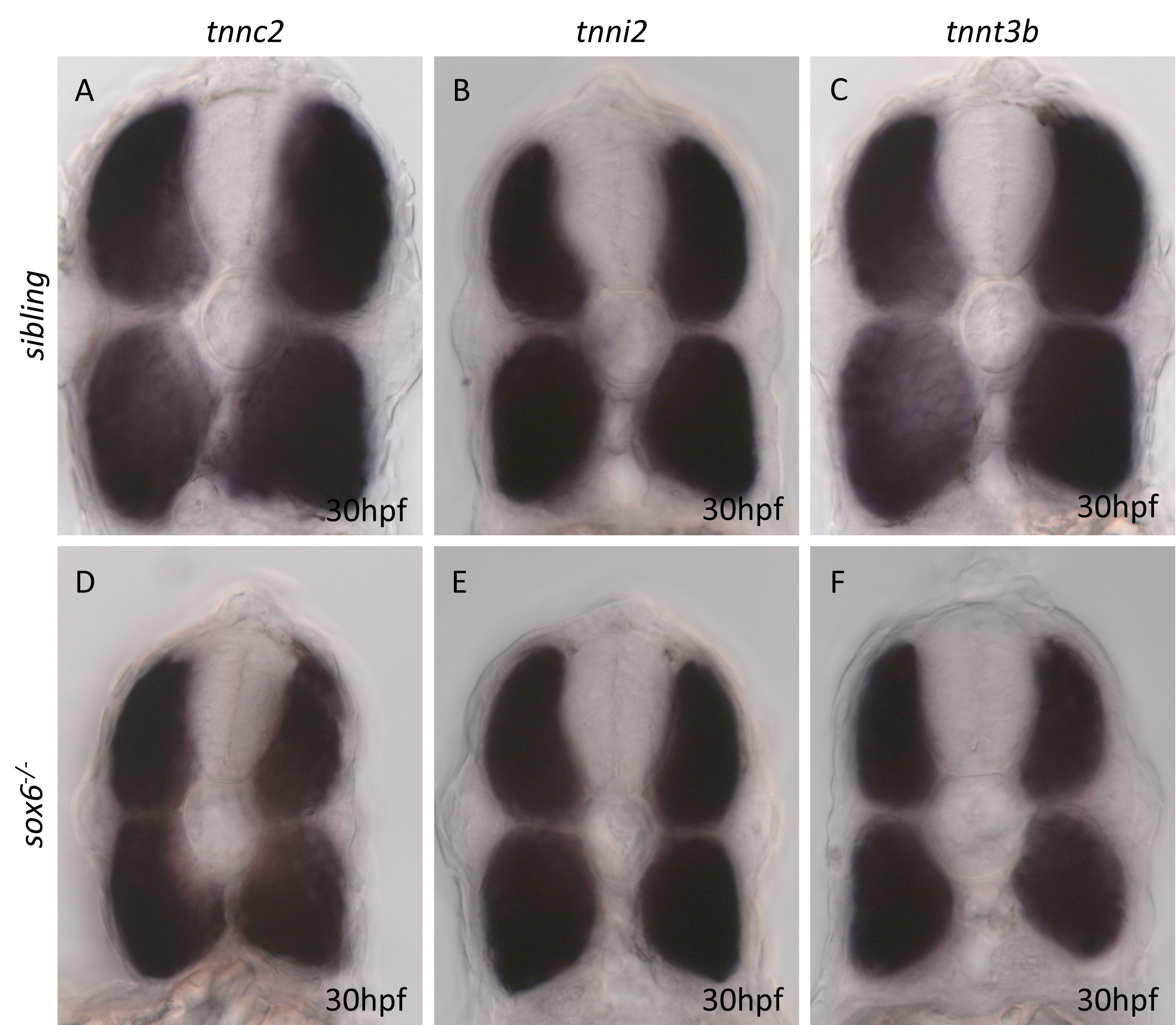

Supplement: Additional file 6: Figure S6. — Expression of fast specific troponin subunit genes in wild type and sox6 mutant embryos. [file 13395_2014_26_MOESM6_ESM.jpeg]

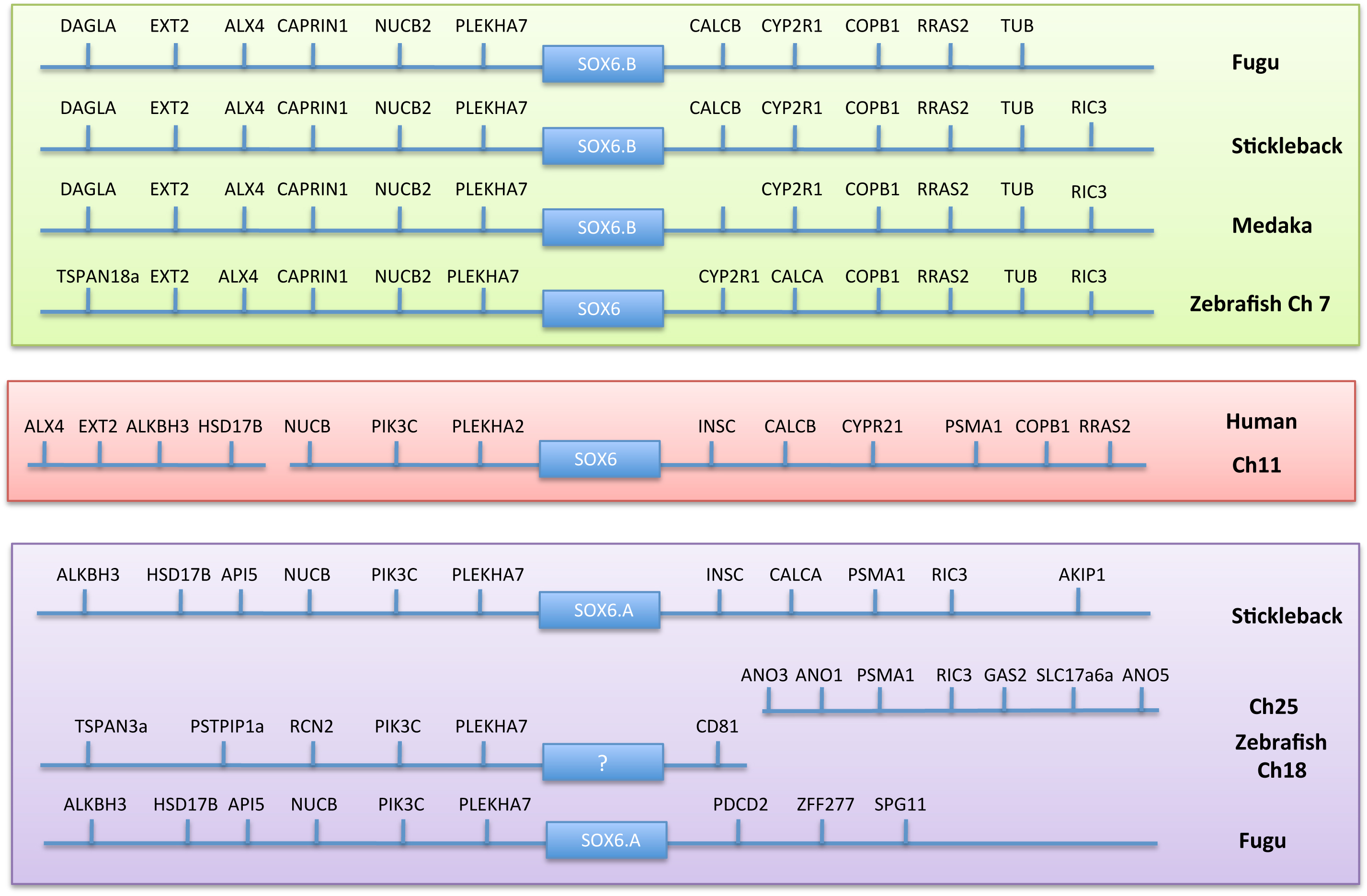

Supplement: Additional file 7: Figure S7. — Chromosomal location of sox6 genes in different fish species and H. sapiens. Two sox6 paralogs are present in the genomes of Fugu, stickleback and Medaka. The sox6B locus in each of these species is located in a chromosomal region that displays conserved synteny with the locations of the zebrafish and human sox6 gene. The sox6A genes of Fugu and stickleback are also located in a region of conserved synteny. This conservation has been lost in zebrafish along with the sox6A paralog (indicated by “?”). [file 13395_2014_26_MOESM7_ESM.jpeg]

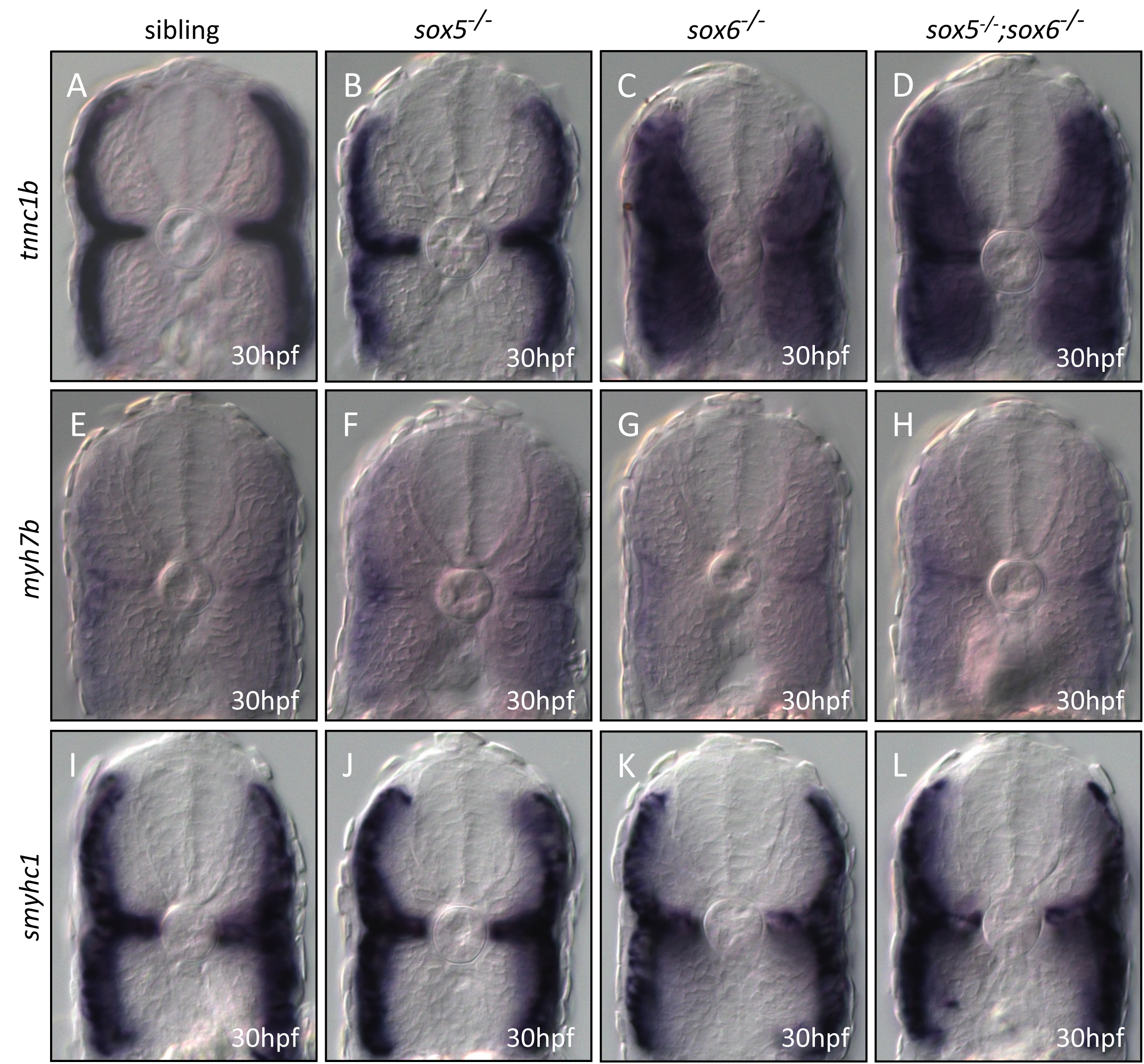

Supplement: Additional file 8: Figure S8. — Expression of smyhc1, myh7b and tnnc1b in wild type, sox5, sox6 and sox5; sox6 double mutants. [file 13395_2014_26_MOESM8_ESM.jpeg]
